# Hepatic ChREBP reciprocally modulates systemic insulin sensitivity in NAFLD

**DOI:** 10.1016/j.jbc.2025.108556

**Published:** 2025-04-29

**Authors:** Aniket Sen, Shilpa Thakur, Priya Rawat, Kajal Jaswal, Budheswar Dehury, Prosenjit Mondal

**Affiliations:** 1 School of Biosciences and Bioengineering, IIT Mandi, Mandi, India; 2 Department of Biological Sciences, Indian Institute of Science Education and Research Berhampur (IISER Berhampur), Berhampur, India; 3 Department of Bioinformatics, Manipal School of Life Sciences, Manipal Academy of Higher Education, Karnataka, India

**Keywords:** ChREBP, insulin sensitivity, PTEN, FGF21, Quercetin

## Abstract

The relation between hepatic ChREBP level and insulin sensitivity remains equivocal. Our study, however, provides compelling evidence that hepatic ChREBP depletion can significantly enhance insulin sensitivity in high-fat and sucrose-fed mice. We have identified that transcriptional induction of hepatic PTEN is driven by ChREBP. Mechanistically, two critical stimuli are elicited in the hepatic ChREBP knockdown condition. The PTEN level is reduced for one stimulus, thereby promoting hepatic insulin sensitivity. The second stimulus, where reduced hepatic PTEN leads to the enhanced release of FGF21, spreads systemic insulin sensitivity. These findings identify hepatic ChREBP as a critical modulator of systemic insulin signaling and suggest that ChREBP downregulation may lead to protection against insulin resistance. Building on this, our molecular dynamics simulation analysis has led to the discovery of a small molecule, Quercetin, that sequesters ChREBP in the cytosol. We report that Quercetin treatment can sequester ChREBP in the cytosol and abrogate high-fat and sucrose-fed–mediated ChREBP nuclear translocation, thereby mimicking the insulin-sensitizing abilities of the hepatic ChREBP knockdown condition. These findings have significant therapeutic implications, suggesting that liver-selective downregulation of ChREBP could protect against systemic insulin resistance that frequently develops early in the pathogenesis of NAFLD and T2DM.

The risk of nonalcoholic fatty liver disease (NAFLD) is rising worldwide due to overconsumption of fat- and carbohydrate-rich diets (1). NAFLD is not only characterized by lipotoxicity resulting from imbalanced *de novo* lipogenesis (DNL) but also by an impaired rate of lipid disposal, coupled with systemic insulin resistance (2). Systemic insulin resistance also contributes to the development of type 2 diabetes mellitus (T2DM) (2).

While DNL is a well-known contributor to NAFLD, the mechanism by which NAFLD leads to T2DM *via* insulin resistance is yet to be fully understood (3). DNL is a highly dynamic biochemical process that involves prominent transcriptional regulators like sterol regulatory element binding protein and carbohydrate response element binding protein (ChREBP) (4, 5, 6, 7, 8, 9). ChREBP has roles in lipid metabolism, gluconeogenesis, fructolysis, and glucose-stimulated insulin secretion, all adding to its involvement in NAFLD development in response to carbohydrates (10, 11, 12, 13, 14, 15, 16, 17, 18, 19).

Although studies have uncoupled ChREBP with insulin resistance for a long time, interestingly, ChREBP has recently been linked with insulin signaling in a contradictory manner. While primary hepatocyte-specific ChREBP overexpression improved insulin sensitivity, a high-fat diet–fed mice model, which also shows increased ChREBP levels, showed insulin resistance (20, 21). Adipose-specific ChREBP KO mice had higher insulin resistance than a fructose-fed control (22). On the other hand, some studies have shown that liver-specific ChREBP inhibition improves systemic insulin sensitivity in ob/ob or high fructose diet–fed mice (23, 24, 25). While these studies fail to reach a common point of agreement, the molecular mechanism by which ChREBP can regulate insulin signaling remains to be understood. The etiology of insulin resistance consists of different determinants that can control complex and multidimensional insulin signaling. Phosphatase and Tensin homolog (PTEN) is one of the postreceptor modulators of the insulin signaling axis (26, 27, 28, 29, 30). PTEN negatively regulates insulin signaling by dephosphorylating phosphatidylinositol 3,4,5-triphosphate (PIP3) into phosphatidylinositol 4,5-bisphosphate (PIP2) (31, 32, 33). Traditionally, PTEN has been defined as a tumor suppressor, as it regulates proliferation, cell growth, and survival to prevent tumor formation (34, 35). However, because the PI3K pathway is a significant signaling network activated in response to insulin, PTEN dysregulation has been implicated in regulating insulin signaling and glucose homeostasis (36, 37). Insulin signaling is initiated with insulin binding to the insulin receptor, resulting in canonical activation of PI3K, an enzyme that phosphorylates membrane-bound PIP2 to PIP3. PIP3, in turn, acts as a docking site for pleckstrin homology domain-containing proteins, which then coordinate the activation of multiple downstream proteins, including AKT/PKB, which in turn then phosphorylates a wide array of downstream targets that initiate the anabolic actions of insulin. PTEN, as a negative regulator of this pathway, dephosphorylates PIP3 to PIP2 to effectively inhibit the effects of PI3K signaling in response to insulin (38, 39, 40). Accordingly, loss-of-function mutations in PTEN can enhance insulin signaling in different organs, leading to protection against insulin resistance, a critical pathogenic process in NAFLD and T2DM (41, 42, 43, 44).

We aim to explore the physiological and pathophysiological relevance of hepatic ChREBP levels in relation to insulin resistance. We depleted hepatic ChREBP in adult mice fed a high-fat-high-sucrose diet (HFSD). We found that the ChREBP knockdown condition leads to PTEN downregulation in the liver. We also delved into the PTEN promoter to elucidate the molecular mechanism of its activation through ChREBP. We understood that ChREBP drives the transcriptional reduction of hepatic PTEN. While hepatic insulin sensitivity is mediated by ChREBP-regulated PTEN, systemic insulin sensitivity in hepatic ChREBP knockdown mice models is modulated by a hepatocyte, fibroblast growth factor 21 (FGF21).

We also investigate whether the downregulation of ChREBP can be mimicked by a small molecule, allowing us to counteract the ChREBP-dependent metabolic alterations. Upon molecular dynamics simulation studies using sorcin–ChREBP interaction as a template, we shortlisted Quercetin as a candidate molecule that can bind to ChREBP. We also tested the ability of Quercetin (QH) to sequester ChREBP in the cytosol and block glucose-dependent nuclear entry of ChREBP. Altogether, our studies support the critical role of hepatic ChREBP in triggering insulin-sensitive signals and explore ways to target ChREBP to curb metabolic syndromes linked with insulin resistance like NAFLD and T2DM.

## Results

### Hepatic ChREBP knockdown improves glucose homeostasis in HFSD-induced obese mice by enhancing insulin sensitivity

**Figure 1:**
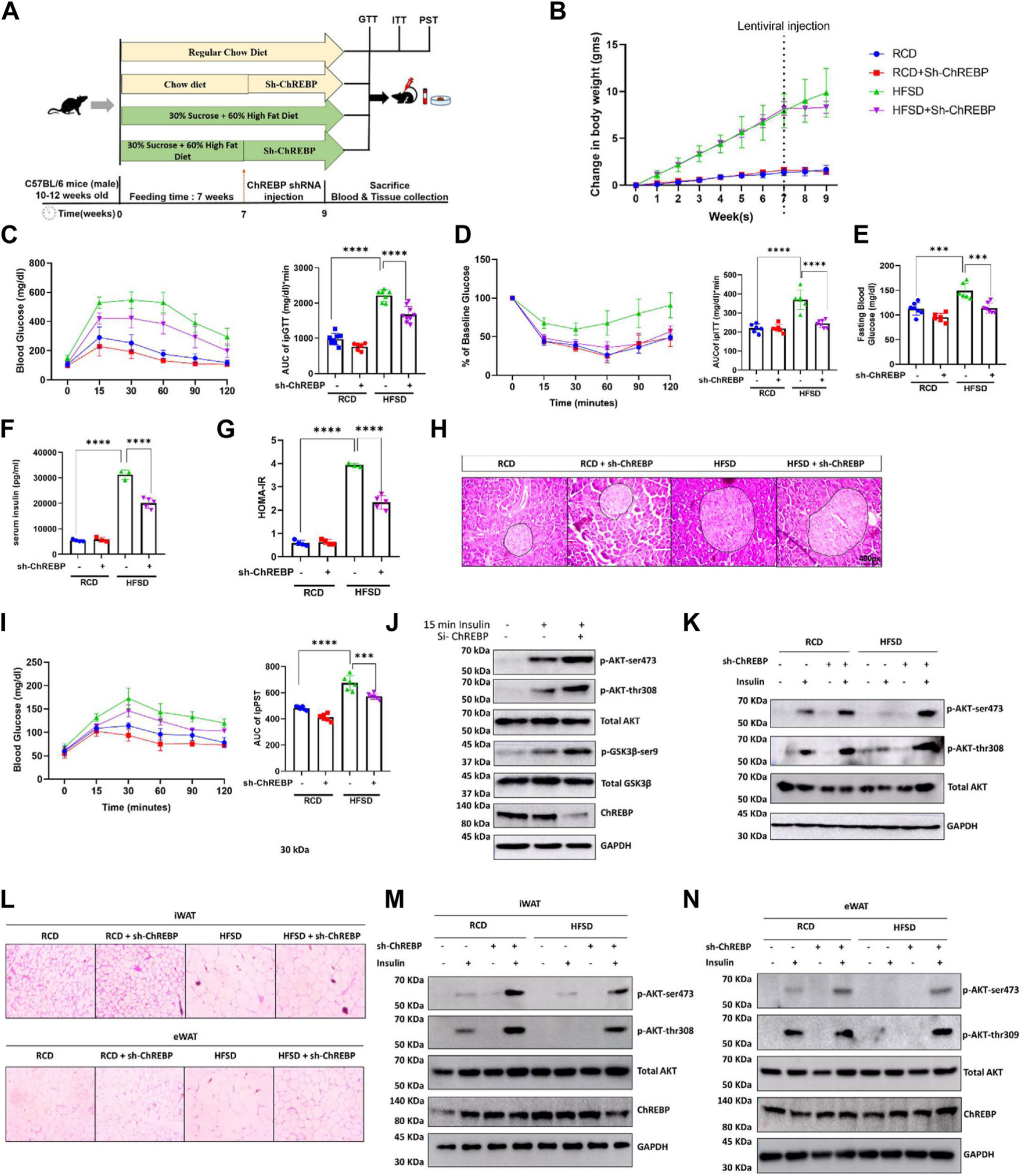
**Hepatic ChREBP knockdown improves systemic as well as hepatic glucose homeostasis in HFSD-induced obese mouse models by enhancing insulin sensitivity.***A*, study plan for the knockout mice study in regular chow diet injected with scramble shRNA (RCD+shScr), ChREBP knockdown in RCD (RCD+shChREBP), 60% fat & 30% sucrose diet–fed mice injected with scramble shRNA (HFSD+shScr) and ChREBP knockdown in HFSD (HFSD+shChREBP). *B*, plot of the change in the body weight of mice from groups described in (*A*) before and after lentiviral injection. *C* and *D*, plot and area under the curve for glucose and insulin tolerance tests after 6 h of fasting for all four groups. *E*, plot of 6-h fasting blood glucose. *F*, plot of the serum insulin levels. *G*, plot showing changes in HOMA-IR, calculated from overnight fasting glucose levels and serum insulin levels. *H*, qualitative H&E staining of a pancreas image taken at 40× magnification. *I*, pyruvate stimulation test after overnight fasting. *J*, immunoblot images and quantification of phosphorylated AKT (serine 473) and AKT (threonine 308), total AKT, phosphorylated GSK (serine 9) and GSK, ChREBP, and GAPDH (used as a loading control). *K*, immunoblot images and quantification of phosphorylated AKT (serine 473), phosphorylated AKT (threonine 308), total AKT, phosphorylated insulin receptor beta tyrosine (1150), total insulin receptor beta, ChREBP, and GAPDH (used as a loading control) from liver lysates of the described mouse groups. *L*, H&E staining of iWAT and eWAT; image captured in 10× magnification showing hyperplasia and adipocyte size. *M* and *N*, immunoblot images and quantification of phosphorylated AKT (serine 473), phosphorylated AKT (threonine 308), total AKT, ChREBP, and GAPDH (used as loading control), from iWAT and eWAT lysates respectively, of the mice groups described in (*A*). (J) is *in vitro* HepG2 cells, and all other images are from in vivo C57BL6 mice models. Mean ± SD. ∗∗∗*p* < 0.001, ∗∗∗∗*p* < 0.0001.

We developed an HFSD obese mice model (Fig. 1*A*) to provide a deeper understanding of the role of hepatic ChREBP in insulin signaling. Ten-week-old C57BL/6 male mice were fed either an HFSD or regular chow diet (RCD) for 7 weeks and then treated with either shScr or shChREBP. We found some protection trends in mice's weight gain after shChREBP injection in the HFSD group. No significant alteration was seen with RCD or HFSD mice’s bodyweight gain after shChREBP or shScr injection (Fig. 1*B*). Remarkably, treating the lean RCD group with shChREBP does not cause hypoglycemia nor does it alter glucose tolerance (Fig. 1*C*) and insulin sensitivity (Fig. 1*D*). Similarly, lean, glucose-tolerant mice treated with shChREBP have similar fasting glucose (FG) compared to shScr-treated RCD mice (Fig. 1*E*). However, after becoming insulin-resistant and obese after 7 weeks of HFSD feeding, mice treated with shChREBP showed marked improvements in GTT (Fig. 1*C*), ITT (Fig. 1*D*), and FG, respectively (Fig. 1*E*) as compared to HFSD+shScr. These results show that shChREBP restores glucose homeostasis in the setting of insulin resistance and obesity. Moreover, we measured fasting serum insulin levels in experimental mouse groups. The RCD+shChREBP mice group did not display any variation in insulin levels compared to the RCD+shScr, whereas the HFSD+shScr group had higher fasting serum insulin levels as expected. However, insulin levels were significantly lowered in HFSD+shChREBP mice (Fig. 1*F*). Similarly, HFSD-fed mice with shChREBP injection having reduced circulating insulin, lower FG, also had an improved insulin HOMA-IR index compared to the HFSD+shScr group (Fig. 1*G*). Following H&E staining of the mouse pancreas, as anticipated, we observed an augmented beta-cell area in the HFSD group, indicating a compensatory response to heightened insulin demand. Interestingly, this increase in the beta-cell area remained comparable between the HFSD+shScr group and the HFSD+shChREBP group, thereby excluding differences in beta-cell mass and size to account for the differences in glucose homeostasis (Fig. 1*H*). shChREBP in HFSD significantly downregulates hepatic glucose production, as evident in the pyruvate stimulation test. It shows that the depletion of hepatic ChREBP in adult mice mediates the restoration of insulin sensitivity, affecting the regulation of hepatic gluconeogenesis in the HFSD+shChREBP group (Fig. 1*I*). Altogether, our findings present *in vivo* evidence supporting ChREBP’s role in modulating the insulin signaling axis.

Furthermore, to understand the role of hepatic ChREBP in insulin signaling *in vitro*, we knocked down ChREBP in HepG2 cells, exposed the cells to 100 nM insulin (HI) treatment for 15 min, and assessed surrogate markers of the insulin signaling pathway. HI treatment given to HepG2 cells in different conditions in various studies indicate that elevated insulin levels can foster insulin resistance (Choubey *et al.*, 2020). We observed that the knockdown of ChREBP increased the phosphorylation of AKT at both serine 473 and threonine 308 residues. We also observed increased phosphorylation of GSK-3β at serine 9 in the ChREBP knockdown condition (Figs. 1*J* and S1, *A*–*D*).

These data suggest that HFSD leads to hepatic and systemic insulin resistance, accompanied by impaired glucose tolerance. Conversely, the downregulation of ChREBP in the liver mediates improvements in systemic glycemia, primarily through the restoration of hepatic insulin sensitivity in HFSD mice.

### ChREBP depletion in the liver of HFSD mice improves insulin sensitivity

Next, we evaluated the impact of hepatic ChREBP downregulation on the insulin signaling axis using mice groups described in Figure 1*A*. To determine the effect of HFSD and depletion of hepatic ChREBP on insulin sensitivity in the liver, insulin (0.5 units/kg body wt.) was injected intraperitoneally into fasted RCD+shScr, RCD+shChREBP, HFSD+shScr, and HFSD+shChREBP mice. pAKT (S473), pAKT (Thr308), and total AKT levels were quantified by Western blots and used as surrogate markers for the insulin signaling axis. Similar levels of pAKT were observed in the RCD+shChREBP mice compared with those in lean RCD mice (Figs. 1*K* and S1, *E* and *F*). However, insulin resistance was observed in the liver of diet induced obese (DIO) mice, characterized by a dramatic reduction in insulin-stimulated pAKT (S473) and pAKT (Thr308) compared to those in lean mice. Interestingly, in HFSD+shChREBP mice, insulin treatment increased pAKT (S473) and pAKT (Thr308) to equal magnitudes to the lean RCD group. Western blotting data revealed that depletion of hepatic ChREBP led to a significant increase in liver insulin signaling in HFSD-fed mice (Figs. 1*K* and S1, *E* and *F*). This indicates that ChREBP is a modulator of the hepatic insulin signaling axis. Our findings also illustrate *in vivo* evidence for ChREBP's negative regulation of liver insulin signaling in HFSD-induced insulin-resistant mice.

It is a relatively well-known fact that insulin resistance contributes to hepatic steatosis. Next, we evaluated the impact of hepatic ChREBP downregulation on intrahepatic lipid accumulation. When fed on RCD, shChREBP treatment did not alter the mice's liver weight gain. However, shChREBP significantly blunted the diet-induced increase in liver weight in HFSD mice (Fig. S2*A*). Histological analysis of the liver H&E section further revealed that the hepatic inflammatory response, hepatic ballooning, microvesicular, and macrovesicular steatosis were ameliorated in HFSD mice treated with shChREBP (Fig. S2*B*). Consistently, HFSD-fed mice exhibited dramatically higher serum AST, ALT, TG, and cholesterol levels, and the accumulation of AST, ALT, TG, and cholesterol levels was decreased by shChREBP in HFSD-fed mice (Fig. S2, *C*–*F*). Furthermore, we found reduced hepatic TG and cholesterol levels in the HFSD+shChREBP mice compared to HFSD mice (Fig. S2, *G* and *H*). We also observed a significant decrease in hepatic lipid droplet accumulation, as indicated by oil-red-o staining, in the ChREBP-depleted group compared to mice feeding an HFSD (Fig. S2*B*). Next, to find the possible molecular mechanisms through which ChREBP depletion attenuates intrahepatic fat accumulation, we measured the impact of ChREBP knockdown on hepatocyte AMP-activated protein kinase (AMPK) activation. Phosphorylation at the Thr172 residue of AMPKα was used as a surrogate marker for its activation. Data suggested that the phosphorylation of acetyl-CoA carboxylase and AMPK were enhanced, and a significant decrease in fatty acid synthase was observed in the HFSD+shChREBP condition compared to HFSD+shScr. (Fig. S2*I*). These data indicate that shChREBP treatment is sufficient to alleviate dyslipidemia and hepatotoxicity.

Upon noticing that hepatic ChREBP depletion in HFSD mice might affect systemic insulin signaling, we examined adipocyte sizes in all four experimental mouse groups. Interestingly, the insulin-sensitive groups (RCD and RCD+shScr) had smaller adipocytes, whereas the HFSD group showed larger average adipocyte sizes. Previous studies have suggested that larger adipocytes may be a predictor of insulin resistance. However, upon hepatic ChREBP knockdown, the size of adipocytes, that is, iWAT and eWAT, was smaller and significantly reduced, suggesting that hepatic ChREBP controls HFSD-induced hypertrophy and reduced hepatic ChREBP level promotes hyperplasia in adipocytes (Fig. 1*L*). Consistent with the size of adipocytes, the relative weight of both eWAT and iWAT was significantly lower in the HFSD+shChREBP group than in the HFSD+shScr group (Fig. S3, *A* and *B*). Additionally, there was no change in the food intake between the HFSD+shScr and HFSD+shChREBP groups (Fig. S3*C*). Our findings indicated that HFSD led to enlarged adipocytes in DIO mice, which may be linked to compromised adipocyte function and metabolism.

Further, to assess the impact of HFSD and knockdown of hepatic ChREBP on insulin signaling in adipose tissue *in vivo*, insulin (0.5 units/kg body wt.) was injected intraperitoneally into fasted RCD, RCD+shChREBP, HFSD, and HFSD+shChREBP mice. pAKT (S473), pAKT (Thr308), and total AKT levels from iWAT and eWAT were quantified by Western blot analysis and used as surrogate markers for adipose tissue insulin signaling. Similar levels of pAKT were observed in the RCD+shChREBP mice compared with those in lean RCD+shScr mice (Figs. 1, *M* and *N* and S1, *G*–*L*). However, insulin resistance was observed in the eWAT and iWAT (Figs. 1, *M* and *N* and S1, *G*–*L*) in DIO mice, with a dramatic reduction in the levels of insulin-stimulated pAKT (S473) and pAKT (Thr308) compared to those seen in RCD mice. Interestingly, in HFSD+shChREBP mice, insulin treatment increased pAKT (S473) and pAKT (Thr308) in almost equal magnitudes to the lean RCD group.

This indicates that liver-tropic restoration of the insulin signaling axis, achieved by downregulating hepatic ChREBP, was sufficient to rescue impaired insulin signaling and hyperplasia in adipose tissue. We found no change in ChREBP level in either adipose tissue, even under shChREBP conditions (Figs. 1, *M* and *N* and S1, *G*–*L*). Consistent with earlier research, our findings indicated that HFSD led to enlarged adipocytes in DIO mice, linked to compromised adipocyte function and metabolism. Depletion of liver ChREBP intervention confirmed that reducing adipose tissue weight and adipocyte size notably improved overall insulin sensitivity. These data suggest that hepatic ChREBP mediates improvements in systemic insulin sensitivity mainly by restoring hepatic insulin signaling in HFSD mice.

### Hepatic ChREBP deficiency reduces PTEN expression

**Figure 2:**
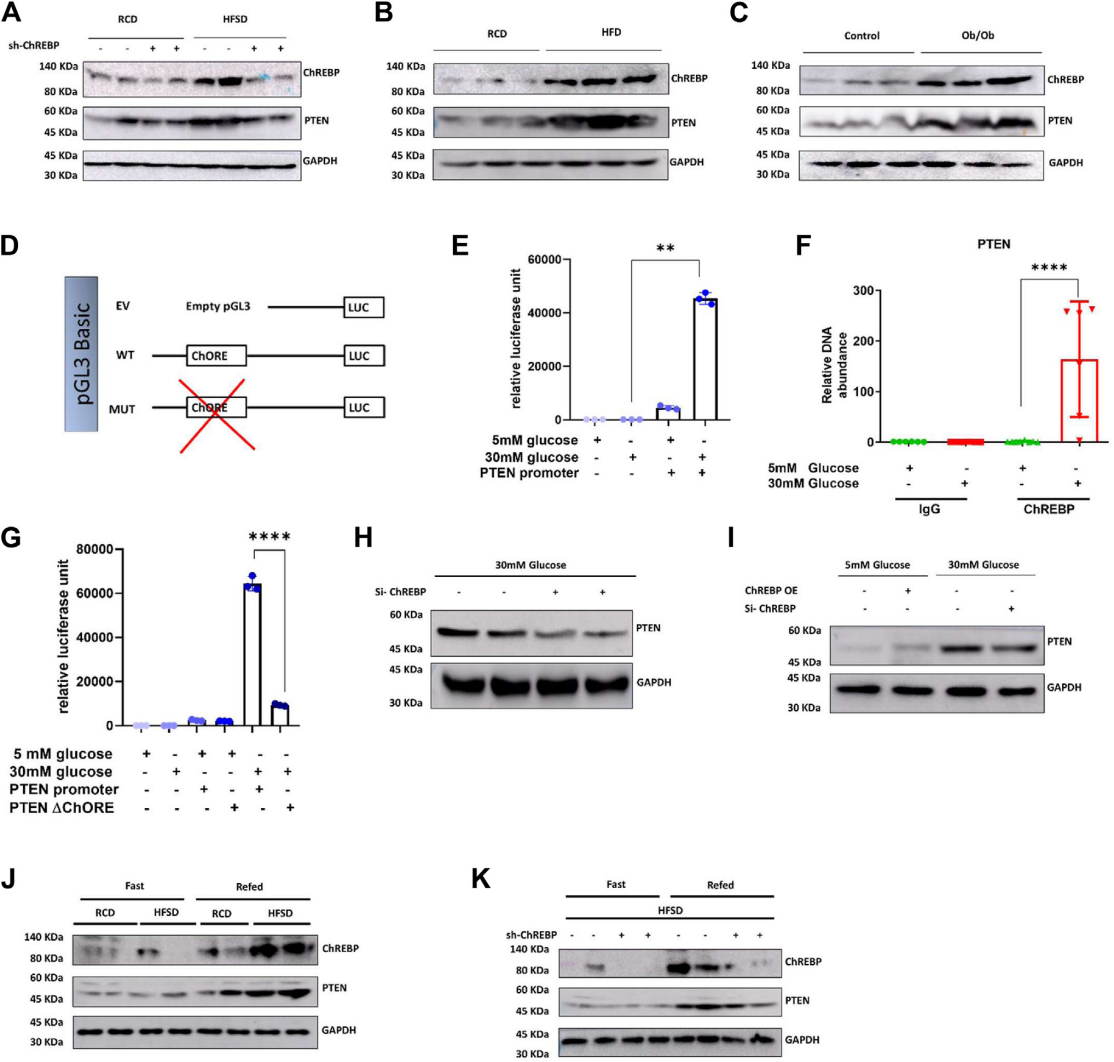
**Hepatic ChREBP deficiency improves insulin sensitivity in DIO mice directly by reducing PTEN expression.***A*, immunoblot images and quantification of ChREBP and PTEN from the liver lysates of all the mice groups discussed in Figure 1*A*, quantified against GAPDH as the loading control (GAPDH used in Figure 1*K* is reused for this blot because the data is from same blot and has the same protein quantification). *B*, immunoblot images and quantification of ChREBP and PTEN, quantified against GAPDH, from liver lysates of mice fed with RCD and 60% high fat diet only (HFD) for 6 weeks. *C*, immunoblot images and quantification of ChREBP and PTEN, quantified against GAPDH, from liver lysates of ob/ob mice against control C57BL/6 mice fed with RCD. *D*, schematic portrayal of PTEN promoter cloned in pGL3 vector having ChORE sequence and the ChORE deletion mutant. *E*, plot of the luciferase assay done in HepG2 cells at low (5 mM) and high (30 mM) glucose concentration after cloning the luciferase construct as shown in (D). *F*, ChIP analysis done in HepG2 cells for PTEN promoter occupancy upon low and high glucose concentration after pulling down with ChREBP antibody. *G*, plot of the luciferase assay at low (5 mM) and high (30 mM) glucose concentration, upon transfection with the WT and MUT into HepG2 cells. *H*, immunoblot image and quantification of PTEN upon ChREBP knockdown in HepG2 cells, quantified with GAPDH as the loading control. *I*, immunoblot image of PTEN upon ChREBP overexpression and knockdown in low and high glucose concentration in HepG2 cells. *J*, immunoblot images and quantification of ChREBP and PTEN in fasting (24 h) and refeeding (24 h) mice liver lysates from mice feeding on RCD and HFSD. *K*, immunoblot images and quantification of ChREBP and PTEN in fasting (24 h) and refeeding (24 h) mice liver lysates of mice given scramble shRNA (HFSD+shScr) *versus* mice with HFSD+shChREBP. *D–I*, are from in vitro HepG2 cells, and all other are from in vivo C57BL6 mice models. Mean ± SD. ∗∗*p* < 0.01, ∗∗∗∗*p* < 0.0001.

Enhanced insulin sensitivity in liver shChREBP mice prompted us to investigate candidate ChREBP’s targets associated with the insulin signaling axis. CHIP-seq analysis revealed that PTEN has a carbohydrate response element (ChoRE) binding site (45). We also observed that hepatic PTEN levels were significantly higher in HFSD mice than in RCD. Interestingly, hepatic ChREBP knockdown led to hepatic PTEN downregulation (Figs. 2*A* and S4, *A* and *B*). Meanwhile, the expression of ChREBP and PTEN were unaltered in both eWAT and iWAT (Fig. S5, *A* and *B*). Based on this observation, we aimed to investigate whether PTEN expression was consistent in other obese mouse models, including those induced by high-fat diet (HFD)-induced obesity or ob/ob mice. Compared to a WT mouse, we saw significant upregulation of both ChREBP and PTEN in the liver of HFD mice (Figs. 2*B* and S4, *C* and *D*) and ob/ob mice (Figs. 2*C* and S4, *E* and *F*).

We next examined whether ChREBP directly regulates hepatic PTEN expression. Through bioinformatic analysis, we came across ChoRE consensuses on mouse PTEN promoters. The mouse PTEN promoter contains one putative functional ChoRE, composed of one consensus CACGTG sequence (46) located at −60 bp upstream of the PTEN transcription start site. We prepared a luciferase reporter plasmid of mouse PTEN promoter with the putative functional ChoRE consensuses found near (−60 to −77 bp) (Figs. 2*D* and S6). A luciferase reporter plasmid containing 100 bp of the mouse PTEN promoter element, transfected into HepG2 cells, showed transcriptional activation in response to high glucose (Fig. 2*E*). We next examined whether ChREBP can directly induce hepatic PTEN expression. ChIP followed by qPCR analysis of the ChoRE revealed a 100-fold enrichment of glucose-dependent occupancy of ChREBP on the PTEN promoter in HepG2 cells (Fig. 2*F*). Further, we used a ChoRE-specific deletion mutant of PTEN promoter (MUT) construct. We observed that cells exposed to high glucose exhibited enhanced PTEN promoter activity in response to high glucose, as long as the ChoRE site remained intact. Mutation of the ChoRE site abrogated the high glucose–mediated transcriptional induction of PTEN (Fig. 2*G*). To further authenticate the association of ChREBP with the transcriptional induction of PTEN, we used siRNA specific to ChREBP and assessed PTEN protein levels in total cell lysates; ChREBP knockdown abrogated high glucose–induced PTEN upregulation (Fig. 2*H*).

In agreement with the ChIP and reporter assay results, the overexpression of ChREBP and high glucose treatment led to robust induction of PTEN expression in HepG2 cells (Fig. 2*I*). Conversely, siRNA-mediated knockdown of ChREBPα in HepG2 cells significantly suppressed high glucose–dependent elevated PTEN expression (Fig. 2*I*). Knockdown was specific, as reflected by RT-PCR, with a significant reduction in ChREBPα expression, but not in ChREBPβ (Fig. S7). The data showed that liver PTEN levels increase in preclinical NAFLD mice models, and ChREBP regulates hepatic PTEN expression.

Having demonstrated the importance of ChREBP as a critical regulator of high glucose–mediated PTEN expression, we aimed to evaluate whether this regulation also exists under physiological conditions. To this end, we investigate the physiological relevance of ChREBP and PTEN expression in a fasted and refeeding state in *in vivo* mouse model. It is well established that the fasting state negatively regulates hepatic ChREBP protein, while the fed state upregulates ChREBP expression. Similar to the reported data, we also observed that mice fasted for 24 h had reduced hepatic ChREBP and concomitant PTEN levels compared with mice fed for 24 h. In contrast, hepatic ChREBP and PTEN levels were upregulated during refeeding (Figs. 2*J* and S4, *G* and *H*). This finding also indicated that the hepatic ChREBP level is controlled by high glucose and hyperinsulinemic cues. Similar to *in vitro* studies, elevated ChREBP may stimulate hepatic PTEN *in vivo*.

To confirm the role of liver ChREBP in mediating refed-induced PTEN expression, we downregulated liver ChREBP using shChREBP in mice on HFSD. The liver lysates from shScr and shChREBP mice were analyzed using a Western blot. shScr mice showed a robust ChREBP expression in the refed state compared to the fasting condition (Figs. 2*K* and S4, *I* and *J*). On a similar line, an induced expression was monitored for PTEN in the refed state. In HFSD+shChREBP mice, in the absence of endogenous hepatic ChREBP, the refed state fails to induce PTEN expression. These findings indicate that glucose levels induce ChREBP’s cytosol-to-nuclear shuttling and stimulate PTEN transcription. Further, *in vivo* disruption of the hepatic ChREBP alone reduces liver PTEN expression even in the presence of physiologically high glucose levels.

### Hepatic ChREBP regulates systemic insulin sensitivity *via* PTEN-dependent FGF21 release

**Figure 3:**
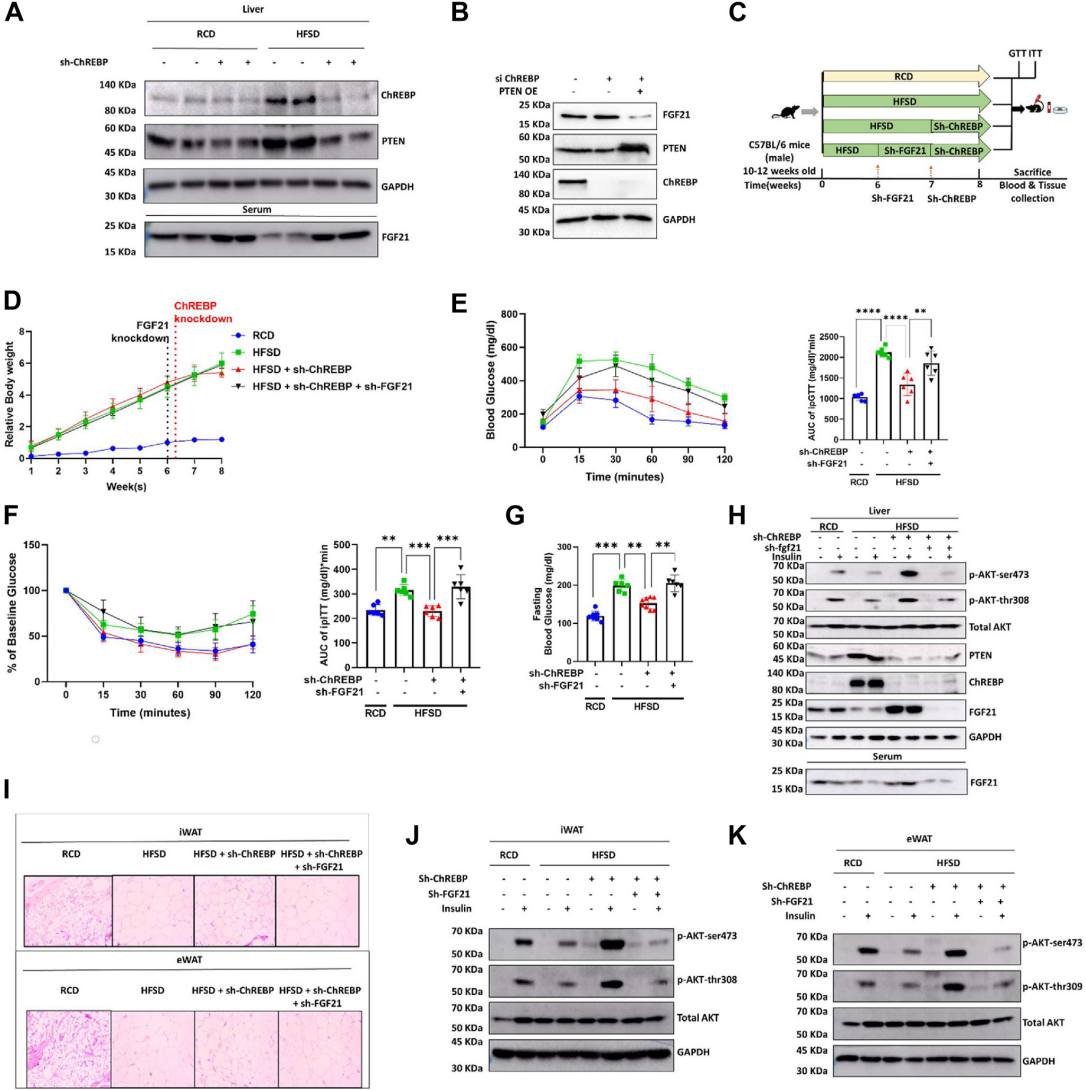
**ChREBP-dependent systemic insulin sensitivity is controlled by FGF21.***A*, immunoblot image and quantification of PTEN, ChREBP, and FGF21 from the liver lysates of the mouse groups described in Figure 1*A* were quantified with GAPDH as a loading control. *B*, immunoblot images of FGF21, PTEN, ChREBP, and loading control GAPDH in ChREBP knockdown and PTEN overexpression conditions in 3T3L1 cells. *C*, study plan for the knockdown mice study in regular chow diet injected with scramble shRNA (RCD+shScr), 60% fat & 30% sucrose diet–fed mice injected with scramble shRNA (HFSD+shScr), ChREBP knockdown in HFSD (HFSD+shChREBP), and ChREBP+FGF21 knockdown in HFSD (HFSD+shChREBP+sh FGF21). *D*, relative body weight of all the groups before and after sh-RNA injections. *E* and *F*, plot and area under the curve for glucose and insulin tolerance tests after 6 h of fasting for all four groups. *G*, plot of 6-h fasting blood glucose. *H*, immunoblot images and quantification of phosphorylated AKT serine (473), phosphorylated AKT threonine (308), total AKT, PTEN, FGF21, ChREBP, and GAPDH (used as loading control) from liver of the mice groups as in (*C*). *I*, H&E staining of iWAT and eWAT; image captured in 10× magnifications showing hyperplasia and adipocyte size. *J* and *K*, immunoblot images and quantification of phosphorylated AKT (serine 473), phosphorylated AKT (threonine 308), total AKT, ChREBP, and GAPDH (used as loading control) from iWAT and eWAT lysates respectively, of the mice groups described in (*C*). *C*, is from *in vitro* 3T3-L1 cells, and rest of the figures are from *in vivo* C57BL6 mice models. Mean ± SEM. ∗∗*p* < 0.01, ∗∗∗*p* < 0.001, ∗∗∗∗*p* < 0.0001.

Liver-derived systemic factors have been reported earlier to affect the metabolism of distal organs. Herein, we explored HFSD+shChREBP mice to identify potential hepatokines that might be responsible for improving systemic insulin sensitivity. Several hepatokines have been shown to enhance metabolic homeostasis in DIO mice (47). The best-characterized example is the liver-derived FGF21 linked with insulin signaling axis (48, 49, 50, 51, 52). To establish the connection, we examined the expression of FGF21 in the serum of RCD+shScr, RCD+shChREBP, HFSD+shScr, and HFSD+shChREBP mice. We found serum FGF21 levels were elevated in the shChREBP group in both RCD and HFD (Fig. 3*A*), which is in agreement with several reports suggesting high systemic FGF21 release in PTEN-deficient mice models (42, 43). To determine if high serum FGF21 release in hepatic ChREBP-depleted mice was PTEN-dependent, we overexpressed PTEN in a ChREBP knockdown condition in HepG2 cells. We found that ChREBP depletion accompanied by PTEN overexpression was unable to upregulate hepatic FGF21 levels (Fig. 3*B* and S8, *A*–*C*). Additionally, to further validate the role of systemic FGF21, we incubated HepG2 and 3T3-L1 cells with serum from experimental mice and checked for lipid accumulation. We performed Bodipy staining to investigate lipid accumulation, finding that cells (HepG2 and 3T3-L1) incubated with serum from the HFSD+shScr group showed higher lipid accumulation than those incubated with serum from the RCD+shScr group. Interestingly, serum collected from HFSD diet mice after ChREBP knockdown (Fig. S9, *A* and *B*) had low lipid accumulation, similar to that of the RCD mice serum group in both HepG2 and 3T3-L1 cells. To understand if the decreased lipid accumulation was due to FGF21, we incubated mice serum with FGF21-neutralizing antibody and treated HepG2 and 3T3L1 cells similarly. FGF21-neutralizing antibody significantly abrogated shChREBP-mediated reduction of intracellular lipid accumulation in both hepatic and adipose tissues (Fig. S9, *A* and *B*), suggesting that FGF21 suppresses HFSD-mediated lipid accumulation.

Our data reveal a novel, indirect mechanism of ChREBP-dependent FGF21 release *via* PTEN, in contrast to several reports suggesting that ChREBP positively regulates FGF21 (53, 54). Here, we found hepatic ChREBP knockdown increased serum FGF21 levels, coinciding with decreased PTEN levels. Our observation is consistent with previous findings linking PTEN knockdown to elevated FGF21 levels (55, 56). Together, our data support that ChREBP regulates FGF21 indirectly through PTEN modulation in the liver, highlighting the complexity of ChREBP–FGF21 interactions.

### FGF21 knockdown in depletion of ChREBP mouse liver fails to improve systemic insulin sensitivity

We next examined the contribution of FGF21 in regulating systemic insulin sensitivity in the context of hepatic ChREBP depletion. To execute this, FGF21 was depleted in the mouse liver by lentivirus (shFGF21), followed by the downregulation of ChREBP (Fig. 3*C*). No change in the gain of body weight between mice of different experimental groups was evident (Fig. 3*D*). Expectedly, depleted ChREBP expression led to the induction of serum FGF21, an effect substantially blunted upon FGF21 knockdown (Fig. 3*H*). Consistently, the shChREBP group showed improved insulin sensitivity, as evidenced by intraperitoneal glucose tolerance test (ipGTT) and intraperitoneal insulin tolerance test (ipITT) among experimental mouse groups (Fig. 3, *E* and *F*). In contrast, the depletion of FGF21 in shChREBP mice substantially reduced systemic insulin sensitivity, as reflected in the ipITT (Fig. 3, *E* and *F*). The fasting blood glucose levels of FGF21-depleted mice were higher than those of the mice with only shChREBP (Fig. 3*G*). These data support the hypothesis that enhanced circulatory FGF21 improves hepatic and systemic insulin sensitivity in ChREBP-depleted mice.

To determine the effect of FGF21 knockdown in ChREBP-depleted mice on insulin sensitivity in classical energy storage tissues such as the liver and adipose, insulin (0.5 units/kg body wt.) was injected intraperitoneally into fasted experimental mice. pAKT (S473) and pAKT (Thr308) were again quantified by Western blots and used as surrogate markers for the insulin signaling axis. As expected, insulin resistance was observed in the liver, iWAT, and eWAT tissues in DIO and HFSD mice, characterized by a dramatic reduction in the levels of insulin-stimulated pAKT (S473) and pAKT (Thr308) compared with those seen in lean RCD mice. Interestingly, in HFSD+shChREBP mice, insulin treatment increased levels of pAKT (S473) and pAKT (Thr308) to equal magnitudes as those in the lean RCD group. Whereas upon depletion of FGF21 in shChREBP + HFSD mice, a blunted response to insulin signaling (at the level of pAKT(S473) and pAKT (Thr308)) was observed in the liver, iWAT, and eWAT, demonstrating classical insulin resistance similar to HFSD group (Figs. 3, *H*, *J*, and *K* and S8, *D*–*L*). We also checked the adipocyte size of all the mice groups. We observed the insulin-sensitive group (RCD) had smaller adipocytes, whereas the HFSD group showed larger average adipocyte sizes. As we observed earlier, hepatic ChREBP knockdown in HFSD resulted in smaller and significantly reduced adipocyte size. Interestingly, the double knockdown of FGF21 and ChREBP resulted in increased adipocytes, as observed in H&E staining (Fig. 3*I*).

Furthermore, we also measured liver ChREBP, PTEN, and FGF21 levels in all experimental mouse groups. Expectedly, HFSD leads to increased ChREBP and PTEN expression, which in turn reduces FGF21 release, while shChREBP leads to the induction of the FGF21 level. We understood that reduced liver ChREBP and PTEN protein levels were associated with elevated serum FGF21 levels. Further, knockdown of the FGF21 expectedly led to decreased serum levels of FGF21 (Fig. 3*H*). Alongside HFSD+shChREBP-FGF21, mice showed significant hepatic lipid accumulation compared to the shChREBP group (Fig. S9*C*). The liver triglyceride and cholesterol content of the HFSD+shChREBP-FGF21 mice was also higher than that of the HFSD+shChREBP group (Fig. S9, *D* and *E*), as evidenced by elevated serum TG, cholesterol, SGOT, and SGPT levels in these mice compared to the shChREBP mice (Fig. S9, *F*–*I*). These observations suggest that HFSD led to the induction of ChREBP-PTEN signaling, thereby reducing serum FGF21 levels and driving systemic insulin resistance. In contrast, the depletion of liver ChREBP resulted in reduced PTEN, which in turn increases hepatic as well as serum FGF21 levels to augment hepatic and systemic insulin sensitivity.

### Quercetin can bind to ChREBP and sequestrate ChREBP in the cytosol

**Figure 4:**
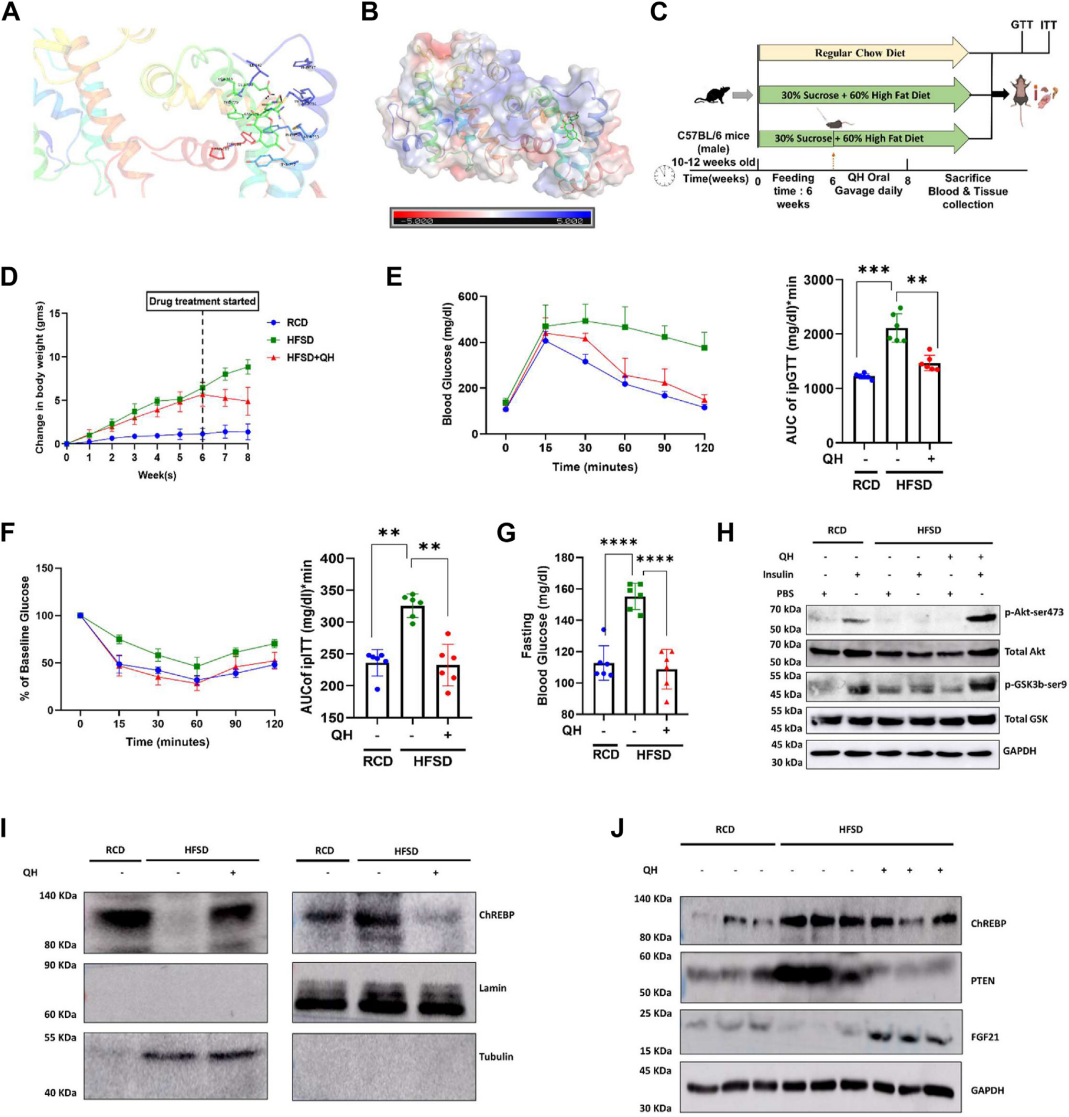
**Quercetin binds to ChREBP and enhances DIO-induced systemic and hepatic insulin sensitivity by downregulating PTEN through the cytosolic sequestration of ChREBP.***A*, structure of the sorcin–ChREBP–QH complex, showing QH interaction with both proteins. *B*, electrostatic potential map of the sorcin–ChREBP–QH complex. *C*, study plan for Quercitrin administration in regular chow diet (RCD), 60% fat & 30% sucrose diet–fed mice (HFSD), and Quercitrin administration in HFSD (HFSD+QH). *D*, relative change in mice body weight before and after QH administration. *E* and *F*, plot and area under the curve for glucose and insulin tolerance tests after 6 h of fasting for all four groups. *G*, plot of 6-h fasting blood glucose. *H*, immunoblot images and quantification of phosphorylated AKT (serine 473), phosphorylated AKT (threonine 308), total AKT, phosphorylated GSK (serine 9), total GSK, and GAPDH (used as a loading control) from liver lysates. *I*, immunoblot images from nuclear cytosolic fractionation of liver lysates probed for ChREBP, tubulin (as cytosolic control), and Lamin ac (as nuclear control) from the described mice groups. *J*, immunoblot images of ChREBP, PTEN, and GAPDH (as loading control) from liver lysates of the mice. All experiments in this figure were conducted *in vivo* using C57BL/6 mice. Mean ± SD. ∗∗*p* < 0.01, ∗∗∗*p* < 0.001, ∗∗∗∗*p* < 0.0001.

Taking cues from mice data on the negative association of hepatic ChREBP transactivation and insulin sensitivity, we next screened for small molecules that can inhibit ChREBP’s nuclear translocation. Our earlier studies have shown that interaction with sorcin can restrict ChREBP in the cytosol and inhibit glucose-dependent nuclear translocation of ChREBP (16). Thus, we used a template of sorcin-ChREBP to find a potential drug molecule that can sequester ChREBP in the cytosol (Fig. S10*A*). Initially, we predicted the 3D structure of ChREBP using alphaFold2 (Fig. S10*B*). Further, we docked the ChREBP protein with already obtained sorcin protein by ClusPro2.0 and refined the structure using HADDOCK (Fig. S10*C*). The electrostatic surface potential map showed the stability of the generated ChREBP–sorcin interaction (Fig. S10*D*). To find a potential drug molecule that can bind to this complex, we turned to the literature and shortlisted five FDA-approved drugs. The interaction of these drugs with the ChREBP–sorcin complex was analyzed using standard precision, extra precision, and molecular mechanics-generalized born surface area (MM/GBSA) scores (Fig. S10*E*). We found that the drug Quercetin (QH) had interaction sites in both sorcin and ChREBP, so we chose to shortlist it. Further, the stability of the generated structure was determined using GROMACS, where we performed molecular dynamics (MD) simulations of both the sorcin–ChREBP and sorcin–ChREBP–QH complexes. The dynamic stability of both complexes was checked by RMS deviation and solvent accessible surface area calculations (Fig. S10*F*). We generated the cluster representative image of the sorcin–ChREBP–QH complex to demonstrate the binding of QH to both proteins (Fig. 4*A*). The electrostatic surface potential map also showed high confidence in the binding of QH to both proteins (Fig. 4*B*). This made us understand that QH might be used as a molecule to sequester ChREBP in the cytosol and mimic the previous ChREBP-knockdown setup.

After identifying QH as having binding ability with ChREBP, we checked whether QH treatment can inhibit glucose-dependent ChREBP nuclear translocation. Immunocytochemistry results showed that the nuclear localization of ChREBP upon high glucose treatment was hindered when the cells were co-treated with QH (Fig. S10*G*).

### Quercetin administration improves glycemic control and insulin sensitivity by sequestering ChREBP in the cytosol

To understand the *in vivo* efficiency of QH, we developed an HFSD obese mice model; ten-week-old C57BL/6 male mice were fed either an HFSD or RCD for 6 weeks and then treated with QH (25 mg/kg body weight) *via* oral gavage for 2 weeks (Fig. 4*C*). We saw a significant decrease in the body weight of HFSD mice upon QH treatment (Fig. 4*D*). Improved glucose tolerance and enhanced systemic insulin sensitivity was observed in QH-treated HFSD mice, as evident on ipGTT, ipITT, and fasting blood glucose level (Fig. 4, *E*–*G*).

Next, we explored the impact of QH treatment in hepatic insulin sensitivity. To check whether the effect of QH treatment could improve hepatic insulin sensitivity, insulin (0.5 units/kg body wt.) was injected intraperitoneally into fasted RCD, HFSD, and HFSD+QH mice. pAKT (S473), pGSK-3β (S9), and total AKT and t-GSK-3β levels were again quantified by Western blots and used as surrogate markers for the insulin signaling axis. Concurrent with our previous observations, significant insulin resistance was observed in the liver of DIO mice, characterized by a dramatic reduction in the levels of insulin-stimulated pAKT (S473) and pAKT (Thr308) compared with those seen in lean mice fed with RCD. Interestingly, QH treatment was able to increase the phosphorylation levels of pAKT (S473) and pGSK-3β (S9) to equal magnitudes as those of the lean RCD group (Figs. 4*H* and S11, *A* and *B*). This data proved that QH oral gavage could help improve HFSD mediated both systemic and hepatic insulin resistance. Next, we aimed to determine if the sequestration of ChREBP was the primary reason for the improved metabolic parameters by performing nuclear cytosolic fractionation on the liver tissue samples from the discussed mice. Consistent with the *in vitro* finding, in liver lysates, we observed a higher abundance of ChREBP in the nucleus of HFSD mice. At the same time, the nuclear localization was significantly inhibited when the mice were treated with QH (Figs. 4*I* and S11, *C* and *D*). QH treatment visibly sequestered ChREBP in the cytosol, thereby impeding its activity as a transcription factor. This observation reinforced that QH treatment can inhibit glucose-dependent transactivation of ChREBP, which mimics the hepatic ChREBP knockdown state. We checked the PTEN and total ChREBP levels from the tissue lysates of the above mice. There was a significant decrease in PTEN levels in mice treated with QH compared to the HFSD-fed controls. Alongside decreased PTEN levels, the QH-administered mice also showed higher hepatic FGF21 levels than HFSD mice, which corresponds to an improved systemic insulin sensitivity as well (Figs. 4*J* and S11, *E*–*G*).

As we have correlated insulin resistance with the progression to steatosis, we also checked the effect of QH administration in DIO mice in terms of DNL. We found decreased lipid droplet accumulation even in high glucose treatment when HepG2 cells were treated with 25 mM QH (Fig. S12*A*). We also saw a significant decrease in lipid droplet accumulation in the liver of QH-treated mice compared to the HFSD control group (Fig. S12*B*). In agreement with *in vitro* data, HFSD-fed mice exhibited drastically higher serum AST, ALT, TG, and cholesterol levels. In contrast, the accumulation of AST, ALT, TG, and cholesterol levels was decreased upon QH treatment in HFSD-fed mice (Fig. S12, *C*–*F*). There was also a significant downregulation of liver triglycerides and cholesterol levels in QH-treated mice compared to the respective diet control of HFSD (Fig. S12, *G* and *H*). Adipocyte size of both eWAT and iWAT significantly decreased upon QH treatment in the HFSD group (Fig. S12*I*), suggesting that QH inhibits HFSD-induced hypertrophy and promotes hyperplasia in adipocytes. We observed a substantial increase in the phosphorylation of acetyl-CoA carboxylase and a significant decrease in fatty acid synthase when HFSD mice were treated with QH *via* oral gavage (Fig. S12*J*). Significantly, there was no decrease in sterol regulatory element binding protein 1C levels in QH-treated groups. The decreased levels of sorcin in HFSD-fed animals were also maintained even after QH treatment (Fig. S12, *K* and *L*). Although there was no change in the relative liver weight in the QH-treated mice, they exhibited specific signs of improved metabolic health, as indicated by a decrease in the relative weight of adipose tissues (eWAT, iWAT), suggesting that QH inhibits HFSD-induced hypertrophy and promotes hyperplasia in adipocytes (Fig. S12*M*). These results demonstrated that pharmacological treatment of QH can prevent high-carbohydrate and fat diet–mediated ChREBP nuclear translocation, thereby attenuating hepatic insulin resistance both *in vitro* and *in vivo*.

## Discussion

Using complementary *in vitro, ex vivo,* and *in vivo* experiments, we identified the liver ChREBP–PTEN axis as a novel modulator of the hepatic and systemic insulin signaling axis. We show that intracellular hepatic stress induced either by treatment with high glucose *in vitro* or by feeding HFSD in mice leads to enhanced expression of ChREBP, which upregulates hepatic PTEN and reduces systemic FGF21 levels. Augmented hepatic PTEN and reduced systemic FGF21 levels, in turn, perturbed hepatic as well as systemic insulin sensitivity, leading to basal hyperinsulinemia and impaired glucose tolerance. Knockdown of hepatic ChREBP leads to reduced PTEN levels in HFSD-fed mice, resulting in improved glycemic control and hepatic and systemic insulin sensitivity, uncovering the liver as a significant contributor to systemic insulin sensitivity. Thus, in HFSD mice, the liver is exposed to two critical stimuli elicited by ChREBP activation: intrahepatic lipid accumulation due to enhanced DNL and PTEN activation, as well as decreased FGF21, which impairs the hepatic and systemic insulin signaling axis. The attenuation of systemic insulin resistance attributed to the PTEN-dependent FGF21 upregulation was also confirmed by deleting FGF21 alongside ChREBP to check the metabolic alterations in DIO mice. We observed that FGF21 knockdown, coupled with ChREBP depletion in the liver, did not significantly improve systemic insulin sensitivity. Furthermore, the results help us understand the previously unknown role of ChREBP in the liver in regulating peripheral organ endocrine signaling to modulate insulin signaling.

We also provided proof that hepatic ChREBP regulates hepatic PTEN expression. PTEN dephosphorylates PIP3 to PIP2 and inhibits the response of P13K to insulin, thereby acting as a negative regulator of the insulin signaling pathway (36, 37, 38, 39, 40). Similarly, studies have shown that PTEN downregulation improves metabolic alterations and glucose homeostasis *via* insulin sensitivity (41, 42, 43, 44). Earlier reports have suggested that PTEN is downregulated in the steatotic livers of obese patients, as well as in the rat models of genetic or diet-induced obesity. However, liver-specific PTEN KO mice (L-PTEN KO) exhibit an ambiguous phenotype. Indeed, with aging, L-PTEN KO mice develop sequentially hepatic steatosis, inflammation, and fibrosis, ultimately leading to hepatocellular carcinoma, indicating that PTEN plays a crucial role in the development of these pathologies (57, 58). Yet, L-PTEN KO mice also exhibit improved glucose tolerance, which is unexpected with NAFLD (55, 56). The effect of hepatic PTEN deficiency on muscle insulin sensitivity and fat storage in adipocytes highly suggests the presence of a crosstalk between the liver and peripheral organs.

Over the past few years, significant efforts have been made to identify and target liver-derived factors to improve metabolic homeostasis in pathophysiological conditions such as obesity, insulin resistance, and T2DM. For this purpose, liver-specific ChREBP-depleted mice may be a helpful model, as these mice exhibit improved adipose tissue insulin sensitivity and reduced white adipose depots and hyperplasia. We further speculate that the release of FGF21 may foster insulin sensitivity of adipose cells by increasing insulin-induced AKT serine phosphorylation. This proof-of-principle experiment, which demonstrates ChREBP knockdown in the liver, has led to improved insulin sensitivity in a peripheral organ, such as eWAT or iWAT, highlighting the liver’s ability to influence the whole body’s metabolic health. This agrees with data supporting the liver as the primary organ of insulin’s action and responsible for glucose and lipid metabolism. In the present study, depletion of liver ChREBP alone was sufficient to restore whole-body insulin sensitivity in HFSD mice. These results support the prevailing role of hepatic insulin sensitivity over that of other tissues in the pathogenesis of metabolic diseases, such as NAFLD, obesity, and T2DM, and highlight the importance of restoring insulin sensitivity–mediated liver metabolism through pharmacological and gene therapy.

Our study reveals a novel, indirect mechanism of ChREBP-dependent FGF21 upregulation mediated by PTEN modulation, which contrasts with the existing literature suggesting direct ChREBP-FGF21 regulation. This indicates that ChREBP regulates FGF21 indirectly through PTEN modulation, highlighting the complexity of ChREBP–FGF21 interactions. Further research is necessary to elucidate the precise signaling pathways, physiological consequences, and promising therapeutic applications of targeting the ChREBP–PTEN–FGF21 axis, underscoring the importance of continued investigation into the molecular underpinnings of metabolic regulation.

QH is a flavonoid used as a dietary supplement (59). We tried to mimic hepatic ChREBP depletion by sequestering ChREBP in the cytosol, thereby limiting its activities as a transcription factor. Upon identifying QH as having binding and sequestration ability for ChREBP, we further elucidated that QH treatment can improve whole-body insulin sensitivity *via* ChREBP. These data suggest an unknown role for ChREBP in regulating the insulin signaling axis and repurposing QH as a cytosolic retention agent for ChREBP. Our studies also indicate that QH can curtail high-fat and carbohydrate diet-induced hepatic and systemic insulin resistance by attenuating the excessive nuclear entry of ChREBP induced by carbohydrates. We strongly advocate ChREBP as a critical hub protein with significant therapeutic potential in insulin resistance–associated metabolic disorders. This novel insight highlights the intricate relationship between ChREBP and metabolic homeostasis, underscoring the need for further investigation into its regulatory mechanisms and potential therapeutic applications.

## Experimental procedures

### *In vivo* study

All the animal experiments were done upon the approval of the IIT Mandi Animal Ethics Committee, the Ministry of Environment and Forest, and the Indian Government. Male C57BL/6 mice (8–10 weeks old) were acquired from IISER Mohali, India. Upon procurement, they were kept in the animal facility of IIT Mandi for 1 week for acclimatization at 25 °C and 50 to 60% humidity, with free access to food and water. They were also kept for acclimatization to 12-h light and 12-h dark cycles for 1 week. Mice were randomly distributed into two groups initially. Group 1 was fed an RCD, which contained ∼14.725% calories from fat, ∼20% proteins, and ∼16.149% carbohydrates (5L79, Lab diet). Group 2 was fed a high fat and high sucrose diet (HFSD), which had 60% calories from fat, 20% carbohydrates and 20% proteins (D12492, Research Diet), and 30% sucrose (HiMedia, PCT0607) dissolved in RO drinking water. Feeding acclimatization was done for six weeks by distributing the mice into two more groups. Four mice from group 1 and group 2 were injected with ChREBP lentivirus. ipGTT and ipITT were performed on the fifth and sixth day of lentiviral injection, respectively. Mice were fasted for 6 h after 2 h of the start of the light cycle. For ipGTT, intraperitoneal glucose injection was given at 2 g/kg body weight. For ipITT, intraperitoneal insulin injection was given at 0.50 IU/kg body weight. ipPST was done on the seventh day of lentiviral injection. For ipPST, mice were fasted overnight and intraperitoneally injected with Na/Pyruvate dissolved in 0.9% saline at 100 mg/kg body weight. For ipGTT, ipITT, and ipPST, blood glucose reading was monitored at 0 min (before injection), 15 min, 30 min, 60 min, and 120 min, respectively. After overnight fasting, mice were sacrificed on the ninth day of the lentiviral injection. Before sacrifice, the fasting blood glucose was monitored. Three mice from each group were injected with insulin (0.50 IU/kg body weight). Serum and tissue samples were collected and snap-frozen immediately for future usage. Liver and white adipose tissue were fixed for further histopathological sectioning in Bouin’s fixative (overnight) and paraformaldehyde (4 h) and washed with 70% ethanol and 30% sucrose, respectively, and stored at room temperature (25 °C) or 4 °C.

### Cell culture and treatment experiments

Most experiments used human hepatocellular carcinoma cells (HepG2) and HEK293T cell lines. One experiment was done using 3T3-L1 cells. All cell lines were bought from ATCC. All the cells were cultured in Dulbecco’s Modified Eagle’s Medium (DMEM High glucose. Invitrogen) and supplemented with 1 mM sodium pyruvate, 10% fetal bovine serum (FBS, Invitrogen, US origin), 199 U/ml penicillin, and 100 μg/ml streptomycin in a 5% humidified CO_2_ incubator. HepG2 cells were given insulin treatment (100 nM) for 15 min before lysis to stimulate insulin signaling. ChREBP knockdown was performed in HepG2 cells using ChREBP-specific si-RNA, using Lipofectamine RNAiMAX Transfection reagent (Thermo Fisher Scientific, 13778075). ChREBP overexpression was done using pChREBP (Addgene, #39235) plasmid, with the help of Lipofectamine 3000 Transfection Reagent (Thermo Fisher Scientific, L3000001). During transfection of both si-RNA and plasmid, cells were kept in reduced serum medium (OPTI-MEM, Thermo Fisher Scientific, 31985062) for 4 h before changing the media to complete DMEM. For low glucose (5 Mm) and high glucose (30 mM) treatments, cells were first normalized with DMEM 5 mM complete media (supplemented with 1 mM sodium pyruvate, 10% FBS (Invitrogen, US origin), 199 U/ml penicillin and 100 μg/ml streptomycin) for 24 h. Then, low- or high-glucose treatments were given for 24 h in a serum-free media.

### p-ChREBP and PTEN overexpression

In OPTI-MEM media, HepG2 cells were seeded in a 6-well plate and transfected with a 5 μg overexpression plasmid (p-ChREBP or PTEN) using Lipofectamine p3000 (1:1 ratio). Four hours after transfection, the OPTI-MEM media was changed to DMEM 5 mM normalization media. After 24 h of transfection, cells were treated with low and high glucose in DMEM serum-free media. After 24 h of transfection, cells were taken out after washing with PBS. Cells were lysed in RIPA lysis buffer, and the lysate was used for further Western blot analysis.

### si-RNA transfection

HepG2 cells seeded in a 6-well plate were transfected with ∼40 nM scramble si-RNA and si-RNA specific for ChREBP using Lipofectamine RNAiMAX in OPTI-MEM media. Four hours after transfection, the OPTI-MEM media was changed to DMEM 5 mM normalization media. After 24 h of transfection, cells were treated with low and high glucose in DMEM serum-free media. After 24 h of transfection, cells were taken out after washing with PBS. Cells were lysed, and the lysate was used for further Western blot analysis.

### Preparation of lentiviral particles

HEK293T cells were used for the preparation of ChREBP lentiviral particles. Cells were transfected with desired shRNAs. Vector containing the desired sh-RNA sequence (AGAAGAGGCGGTTCAATATTA) for mouse Mlxipl (targeting both ChREBP α and β canonical isoforms); (CTCTACACAGATGACGACCAA) for mouse FGF21 (both validated constructs), were o-transfected with the help of two lentiviral packaging vectors psPAX2 (Addgene, #12260) and pMD2.G (Addgene, #12259). After concentrating, the particles were injected *via* the tail vein at a concentration of ∼10^12^ pfu/mice to achieve liver-specific knockdown.

### Promoter luciferase assay

The ChoRE-binding site on the PTEN promoter was identified manually. Primers were designed for cloning the sequence from the genomic DNA of mice (FP- CGGGGTACCTTTCCGAGGCGCCCTGCT; RP- CCGCTCGAGATGGCTGCAGCTTCCGA). The ChoRE-binding site of the promoter was cloned into the PGL3-basic backbone luciferase as a marker. The cloned product was transfected in HepG2 cells, and later trypsinized for low and high glucose treatment after shifting into a 96-well plate in triplets of each condition (10,000 cells/well). Upon 24 h of respective nutrient treatment, cells were lysed using Bright-Glo luciferase (Promega, E2610), and their luciferase activity was measured in the Tecan multiplate reader.

### Chromatin immunoprecipitation

HepG2 cells were seeded in 10 cm and shifted to low (5 mM) and high (30 mM) glucose conditions as described before. Chromatin immunoprecipitation was done using the ChREBP antibody (Abclonal, A7630) and q-PCR primers for the ChoRE-binding site on the PTEN promoter (FP-TTTCCGAGGCGCCCTGCT; RP-ATGGCTGCAGCTTCCGA). The manufacturer’s protocol mentioned in the Actif motif CHIP-IT EXPRESS kit (53008) was followed for further assay and analysis.

### Bodipy staining

As mentioned, HepG2 cells were seeded on coverslips and treated with high and low glucose. Twenty five micromolars of QH dissolved in 100% ethanol was treated for 24 h. Cells were fixed in 4% parafolmaldehyde before 2 μM of Bodipy staining for 30 min. Coverslips were mounted with DAPI mounting media and visualized in a confocal microscope.

### Immunocytochemistry

As mentioned, HepG2 cells were seeded on coverslips and treated with high and low glucose. Twenty five micromolars of QH, dissolved in 100% ethanol, was treated for 24 h. Cells were fixed in chilled methanol, followed by PBST washing and blocking in PBST supplemented with FBS. Overnight primary antibody (1:1000) incubation was done at 4 °C, followed by washing and 2 h of secondary antibody (1:500) incubation. Cells were mounted with DAPI mounting media and visualized in a confocal microscope.

### Subcellular fractionation

The harvested tissue was used for nuclear and cytosolic fractions. Following the manufacturer's protocol, nuclear and cytoplasmic fractions were isolated using the nuclear and cytoplasmic extraction kit (Thermo Fisher Scientific, 78833). The proteins from both fractions were quantified using bicinchoninic acid assay. Lamin ac and tubulin were used as control for nuclear and cytoplasmic fractions, respectively.

### Western blot analysis

Tissue fractions were subjected to PBS wash at 500*g* before lysis in RIPA lysis buffer (added with 1× protease and phosphatase inhibitor). Lysis was done strictly on ice with occasional vortexing for 30 min. Centrifugation was done at 15,000 rpm for 15 min to collect the proteins as supernatant and pellet out the debris. The bicinchoninic acid assay protocol (Thermo Fisher Scientific, 23227) was followed to quantify the proteins. Protein was loaded in an SDS Page for gel electrophoresis and was later transferred onto a nitrocellulose membrane. The blots were blocked in 5% skimmed milk at room temperature for 1 h. Blots were then probed against specific primary antibodies overnight at 4 °C. All the antibodies were used in 1:2000 dilution. After overnight incubation, the blots were washed in 1× TBST thrice for 10 min before incubating in specific Horseradish peroxide–conjugated secondary antibodies (mouse/rabbit) in 1:3000 dilution for 1 h. They were visualized *via* chemiluminescence (ECL, Bio-Rad, 1705060).

### Quantitative PCR

The snap-frozen liver samples or cells seeded in 6 well plates, were used for RNA isolation using RNA-Xpress reagent (Himedia). The rest of the RNA extraction procedure was done manually using chloroform-isopropanol. One microgram of RNA was used for complementary DNA preparation using the iScript complementary DNA synthesis kit (Bio-Rad). The gene expression analysis was done using SYBR Green (Bio-Rad) in a real-time thermomixer (Bio-Rad). 18s was used as a housekeeping gene, and the analysis was done using the comparative cycle threshold (Ct value) method.

### Histopathological analysis

Tissue samples collected in Bouin’s solution were used for paraffin sectioning, and the samples collected in 4% paraformaldehyde were used for cryosectioning. For histopathological analysis, paraffin sections were used for H&E staining, while the cryosections were used for oil red-O staining to evaluate lipid accumulation.

### Isolation of fat from liver tissues

Frozen liver samples (∼50 mg) were taken out, weighed, and washed in PBS. Ten volumes of ice-cold PBS were used to homogenize the samples. Forty percent of the homogenate was transferred to a new tube before adding 1.2 ml chloroform:methanol (2:1, v/v). They were mixed by vigorous vortexing for ∼30 s. The samples were centrifuged at 4200 rpm for 10 min at 4 °C after adding 100 μl of ice-cold PBS. 0.2 ml of the organic phase (bottom layer) was transferred into a new tube for evaporating with the help of a speed-vac. The dried lipids were then dissolved in 200 μl of 1% Triton X-100 and used for the measurement of triglyceride (Sigma-Aldrich, MAK266) and cholesterol (Abcam, AB65359) content by strictly following the manufacturer’s protocol.

### Analysis of biochemical parameters

The blood samples collected during mouse sacrifice were subjected to serum isolation by centrifugation at 1000 rpm for 5 min. The serum samples were then analyzed for aspartate aminotransferase (Sigma-Aldrich, MAK055), alanine transaminase (Sigma-Aldrich, MAK052), cholesterol (Abcam, AB65359), and triglycerides (Sigma-Aldrich, MAK266) strictly following the manufacturer’s protocol.

### Molecular modeling

Due to the lack of experimental structure of ChREBP, we sought to predict the three-dimensional structure using homology modeling and the machine-learning approach deployed in AlphaFold2. Due to a lack of suitable templates for modeling ChREBP in RCSB PDB, we opted for a machine-learning modeling approach. The in-house–deployed AlphaFold2 was used to predict the structure, where we observed the C-terminal domain of the highest predicted local distance difference test score, which signifies the model's confidence. Additionally, various standard protein model evaluation servers, including PROCHECK, ERRAT, ProSA, ProQ, and MolProbity, were utilized to evaluate the stereo-chemical qualities of the modeled domain of ChREBP. The high-resolution experimental x-ray crystallographic structure of sorcin (UniProt: P30626) was obtained from PDB (PDB ID: 4USL).

### Protein-protein docking

Molecular docking is highly effective in generating numerous potential protein–protein complex models. ClusPro2.0 was initially deployed to default blind docking ChREBP with sorcin without restraints. The highest-ranked cluster, characterized by the most significant number of representatives with the lowest energy, was selected for further refinement through HADDOCK 2.4. In HADDOCK, the interacting atom pairs of ChREBP and sorcin derived from the top-ranked ClusPro cluster were designated "active residues." In contrast, "passive" residues were automatically identified in the surrounding area before the docking submission. Unlike *ab initio* docking methods, HADDOCK incorporates the docking procedure in three phases: random orientation generation, energy minimization to resolve steric clashes, and torsion angle dynamics with torsion angles as variables, followed by refinement in Cartesian space with explicit solvent. HADDOCK deemed the top cluster exhibiting the lowest z-score the most reliable. It was subsequently examined for intermolecular interactions and optimized through all-atom MD simulations.

### Molecular docking of natural compounds against ChREBP–sorcin complex

#### Preparation of ligands

To understand how natural compounds interact with the ChREBP–sorcin complex, we obtained their 3-D structures from the PubChem database and saved them in SDF file format. Prior to docking, all compounds underwent energy minimization using the Ligprep module of Schrodinger with an OPLS3e force field until an energetically stable conformation was achieved. The ligands were prepared with a pH of 7.0 ± 2.0, with default parameters selected for generating tautomers and possible states of the molecules.

#### Docking

To enhance the hydrogen bonds and ensure appropriate charged states for the ionizable amino acid residues, the ChREBP–sorcin complex obtained from the top-ranked cluster of HADDOCK, we used the Protein Preparation Wizard of Maestro with a pH of 7.0, determined with the PROPKA module. The PointSite model was then utilized to identify the binding site residues within the ChREBP–sorcin complex. Subsequently, the receptor grid was generated by specifying active site residues using Maestro 12.8. A total of five natural compounds were screened. The PrankWeb machine-learning approach was also employed to validate the binding site residue prediction. To assess the efficacy of natural products, the Glide docking program in the Schrodinger suite was utilized to study the molecular interactions with the ChREBP–sorcin complex. The docking calculations were initially carried out in standard precision mode and then in extra precision mode. The resulting F13–polyphenol complexes were then visualized using PyMOL (http://www.pymol.org/pymol) and LigPlot+.

#### MM/GBSA calculations

The MM/GBSA method, as implemented in Prime by Schrödinger, LLC in New York in 2021-2, was utilized to forecast the binding free energy (ΔG_bind_) of the natural compounds. Then, we evaluated the stability of the quercetin complex through all-atom MD simulations.

#### All-atoms MD simulations

After conducting docking studies, the ChREBP–sorcin–quercetin complex and ChREBP–sorcin systems were subjected to MD simulations studies to assess the structural dynamics stability of both the systems with the CHARMM36m force field in GROMACS, utilizing the TIP3P water model. The ligand topologies were created using the CHARMM General Force Field, and 0.15 M NaCl was introduced to each system for neutralization purposes. Following electro-neutralization, energy minimization was carried out using the steepest descent method for 5000 steps, and subsequent equilibration was performed in NVT and NPT ensembles with periodic boundary conditions. The systems were maintained at a constant pressure of 1 bar and temperature of 303K during a 200 ns production MD run, employing the Parrinello-Rahman barostat algorithm for simulation.

### Trajectory analysis

Both systems' trajectories were harvested and corrected for the removal of periodic boundary condition effect by utilizing tools from the GROMACS package. The dynamics stability and conformational flexibility were measured by computing the backbone RMSD and RMSF using gmx rms and gmx rmsf tools. Additionally, solvent accessible surface area and radius of gyration (Rg) were determined with gmx sasa and gmx gyrate tools, and changes in secondary structure were analyzed using the gmx do dssp tool for both the systems. Hydrogen bond formation was examined with gmx hbond tool, and visualization was achieved through VMD and PyMOL software, with graphical representations generated using XmGrace Software.

### Statistical analysis

All the data represented are as mean ± SD. All the statistical analyses were performed in GraphPad Prism 8 software. ANOVA was used to compare two or more groups; we used unpaired t-tests and one-way ANOVA, respectively, followed by the Bonferroni *post hoc* test. Analysis with a *p*-value < 0.05 was considered to be significant.

## Data availability

The manuscript includes all study data and supporting information.

## Supporting information

This article contains supporting information.

## Conflicts of interest

The authors declare that they have no conflicts of interests with the contents of this article.

## Supporting information

Supplemenatary Material
