# Supplementary material for: Hepatic ChREBP reciprocally modulates systemic insulin sensitivity in NAFLD
Source: J Biol Chem. 2025 Apr 29;301(6):108556. doi: 10.1016/j.jbc.2025.108556 (PMC12155594; doi:10.1016/j.jbc.2025.108556)
Supplement: Supplemenatary Material [file mmc1.docx]

**Hepatic ChREBP reciprocally modulates systemic insulin sensitivity in NAFLD**

Aniket Sen^1^, Shilpa Thakur^1^, Priya Rawat^2^, Kajal Jaswal^1^, Budheswar Dehury^3^, Prosenjit Mondal^2*^

^1^ School of Biosciences and Bioengineering, IIT Mandi, Mandi, India **^2^**Department of Biological Sciences, Indian Institute of Science Education and Research Berhampur (IISER Berhampur), Berhampur 760010, India, ^3^Department of Bioinformatics, Manipal School of Life Sciences, Manipal Academy of Higher Education, Manipal-576104, India

^*^Corresponding author

Corresponding authors: [pmondal@iiserbpr.ac.in](mailto:pmondal@iiserbpr.ac.in).

**Supplementary Figures**

**
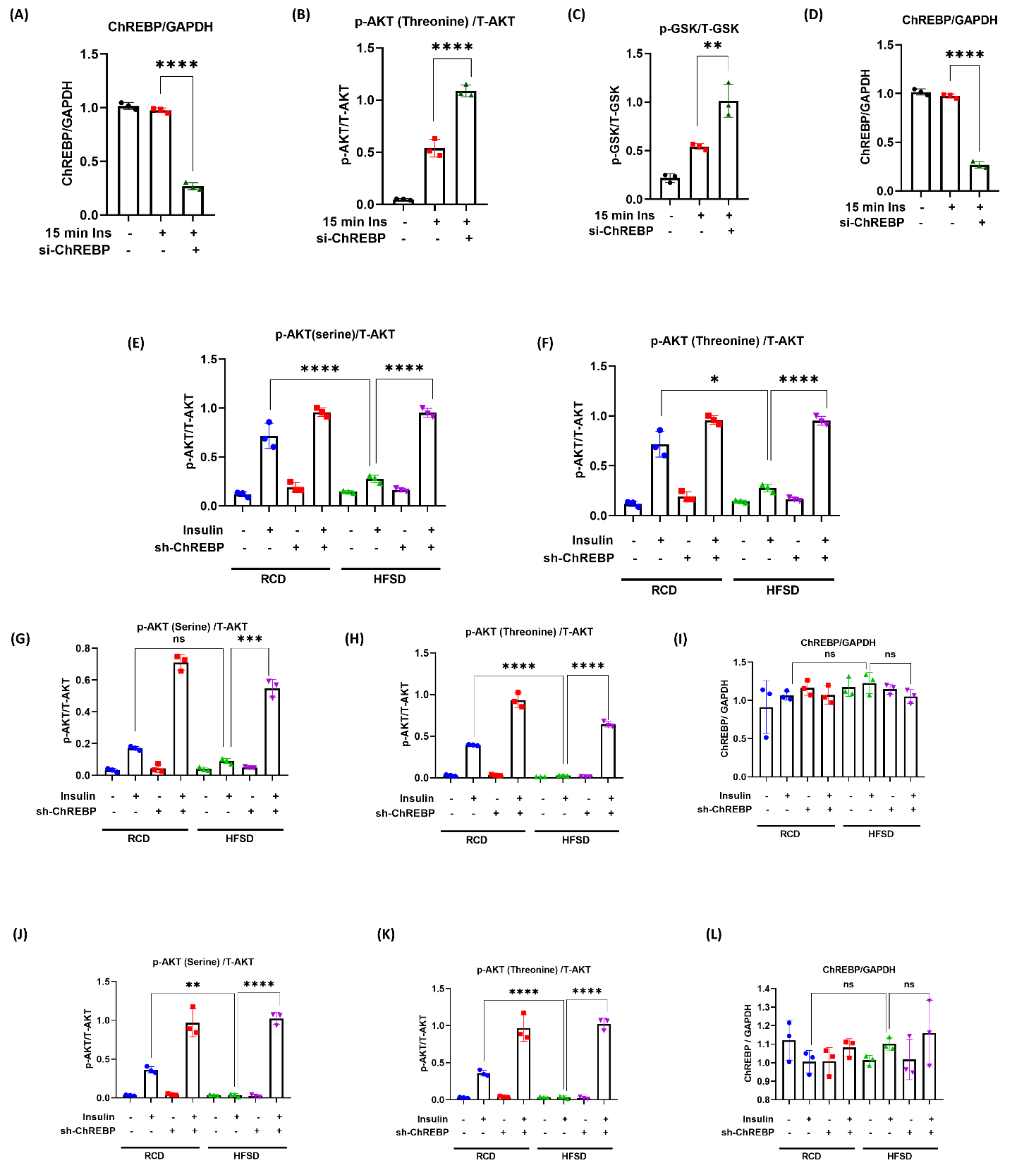
**

**Figure Supplementary 1: Quantification of Western blot images of Figure 1.** (A-D) Quantification of Figure 1J. (E-F) Quantification of Figure 1K. (G-I) Quantification of Figure 1M. (J- L) Quantification of Figure 1N. Mean±SD.***P* < 0.01, ****P* < 0.001, *****P* < 0.0001.


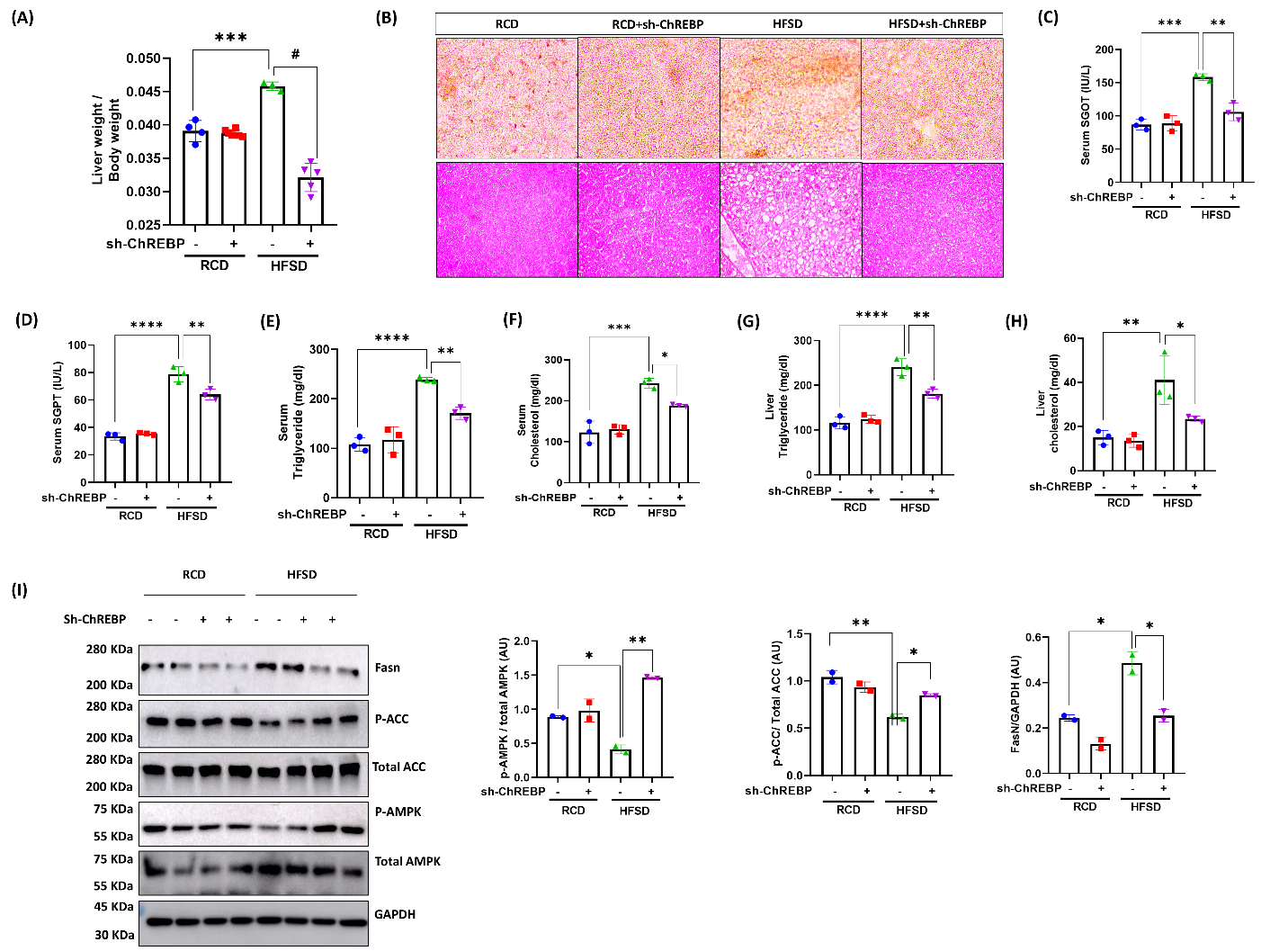


**Figure Supplementary 2: Hepatic ChREBP depletion attenuates DNL.** (A) The plot of liver weight for all four groups was normalized by the respective body weight of the mice. (B) ORO (top) and H&E (bottom) staining images of liver tissue, taken at 10X magnification. (C, D, E, F) Plot the serum parameters, including SGOT, SGPT, cholesterol, and triglycerides, for all four groups. (G, H) Plot of the liver triglyceride and cholesterol. (I) Immunoblot images and quantification of Fatty Acid Synthase (FASN), phosphorylated ACC (serine 79), ACC, phosphorylated AMPK (threonine 172), AMPK, and GAPDH (used as a loading control) from the same mouse groups. All experiments from this figure are conducted in-vivo from C57BL6 mice. Mean±SD. **P* < 0.05, ***P* < 0.01, ****P* < 0.001.


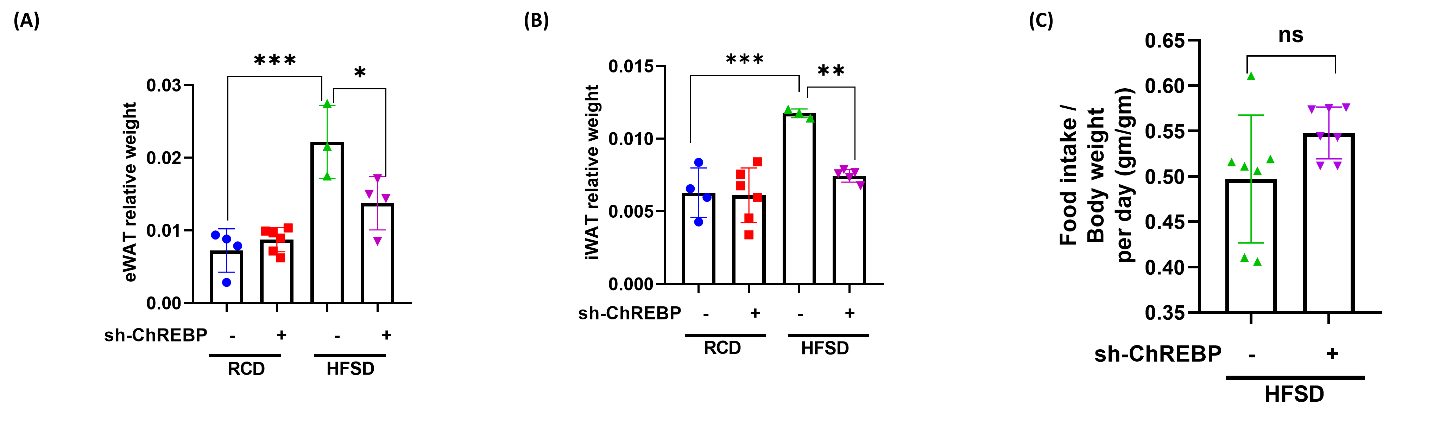


**Figure Supplementary 3: Hepatic ChREBP depletion decreased WAT weight in DIO mice.** (A, B) Relative eWAT and iWAT weight normalized with body weight for RCD+shScr, RCD+shChREBP, HFSD+shScr and HFSD+shChREBP. (C) Food consumption of mice feeding on HFSD was plotted by normalizing with body weight (data collected for seven days). All experiments from this figure are conducted in-vivo from C57BL6 mice. Mean±SD. ns = non-significant, **P* < 0.05, ***P* < 0.01, ****P* < 0.001.


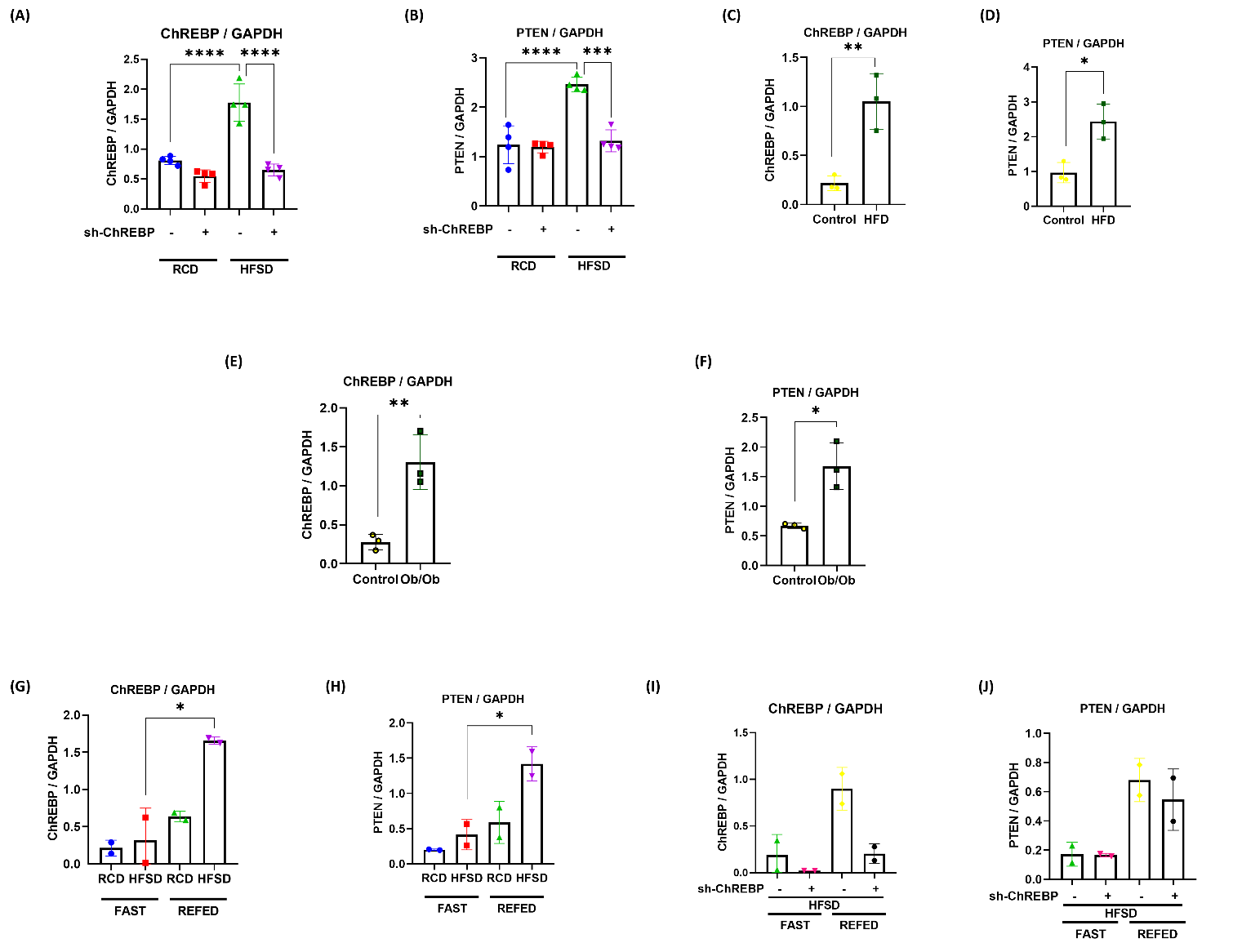


**Figure Supplementary 4: Quantification of Western blot images of Figure 2.** (A-B) Quantification of Figure 2A. (C-D) Quantification of Figure 2B. (E-F) Quantification of Figure 2C. (G-H) Quantification of Figure 2J. (I-J) Quantification of Figure 2K. Mean±SD. **P* < 0.05, ***P* < 0.01, ****P* < 0.001, *****P* < 0.0001.


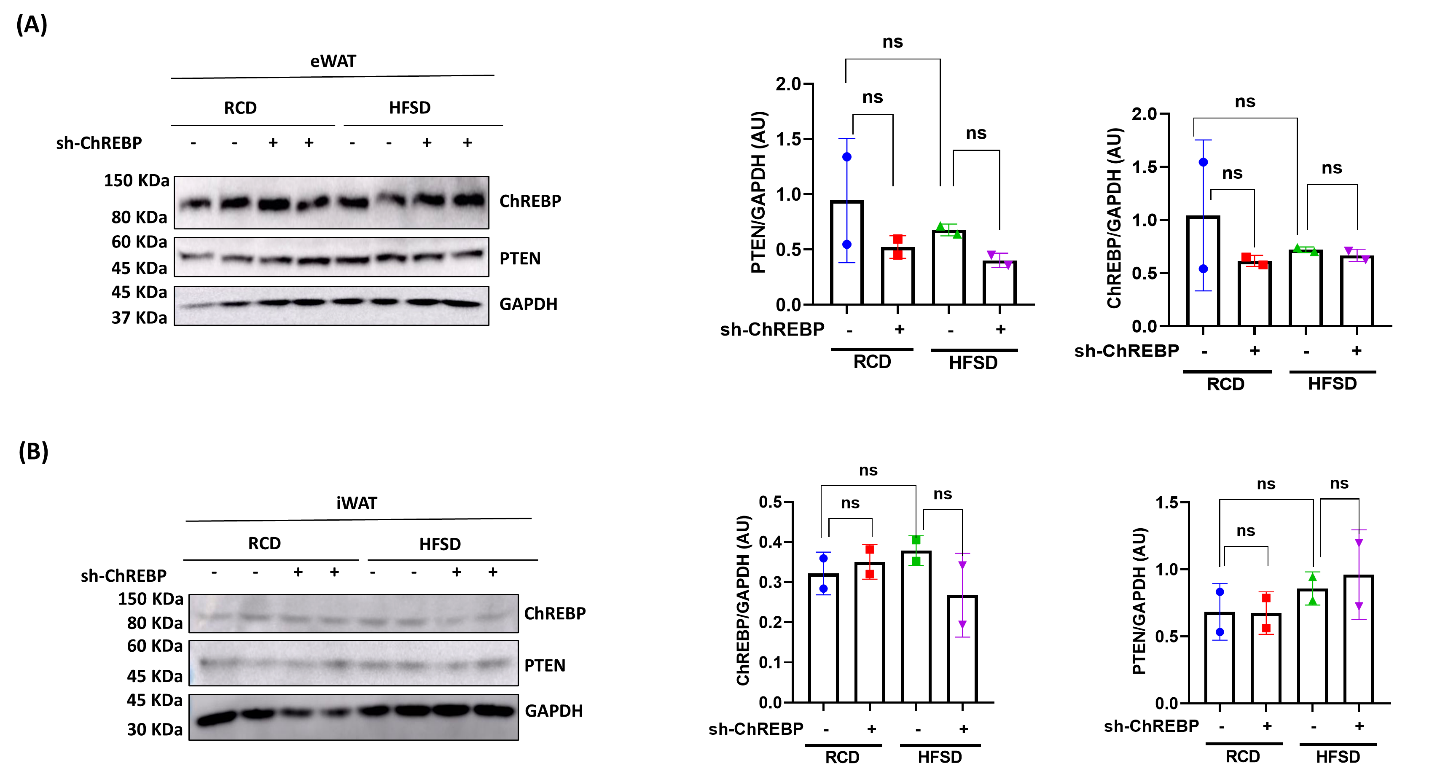


**Figure Supplementary 5: Expression of ChREBP and PTEN in iWAT and eWAT of sh-ChREBP mice remains unchanged.** (A-B) Immunoblot images and quantification for ChREBP and PTEN protein expressions in eWAT and iWAT, normalized by GAPDH as loading control from RCD+shScr, RCD+shChREBP, HFSD+shScr and HFSD+shChREBP groups. All experiments from this figure are conducted in-vivo from C57BL6 mice. Mean±SD. ns = non-significant.


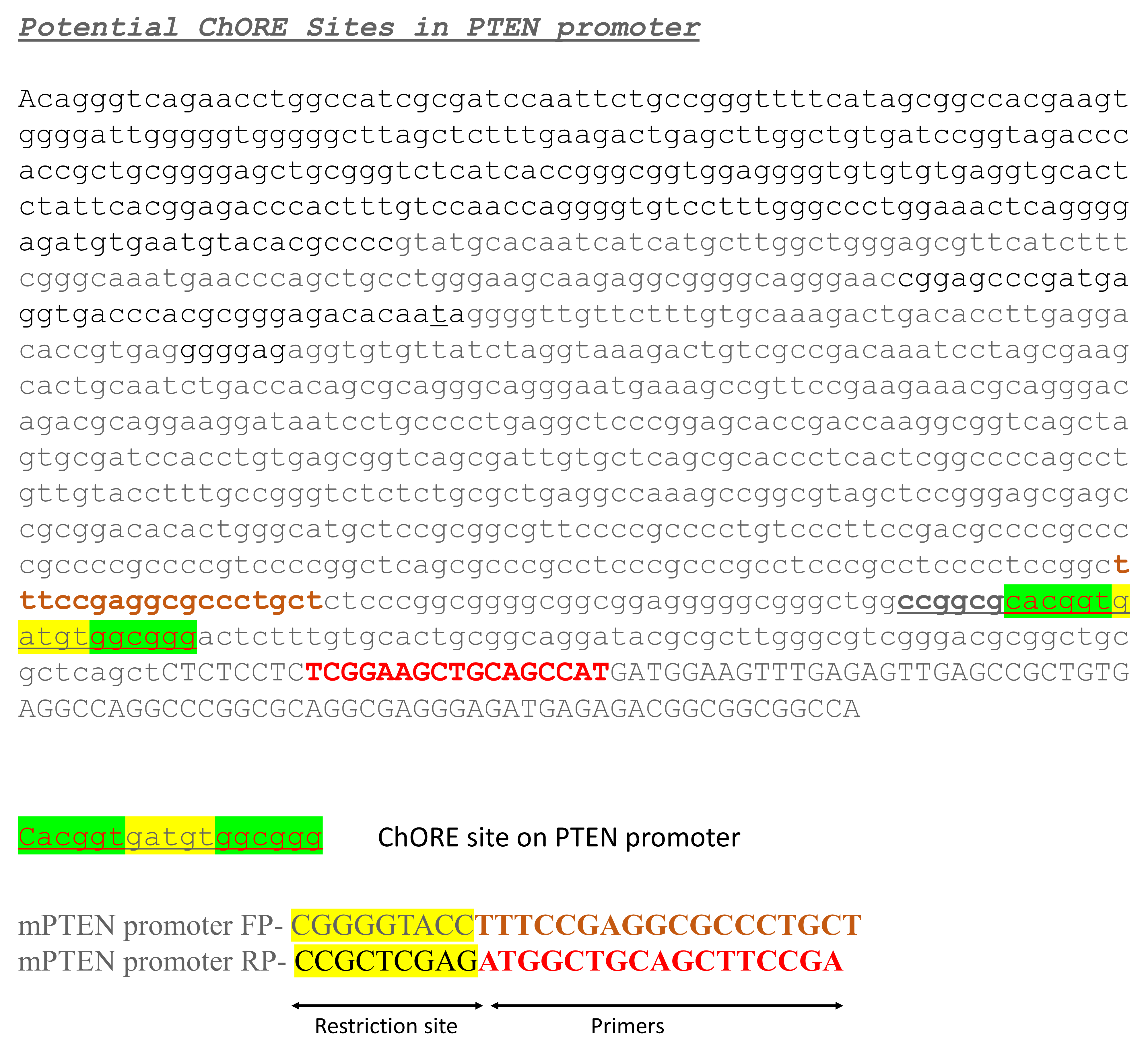


**Figure Supplementary 6: ChoRE binding site present on PTEN promoter**

**Figure Supplementary 7: Relative mRNA levels of ChREBP isoforms upon si-ChREBP knockdown.** RT-PCR to measure the expression levels of both ChREBP isoforms upon si-ChREBP knockdown in HEPG2 cells. Mean±SD. *****P* < 0.0001.


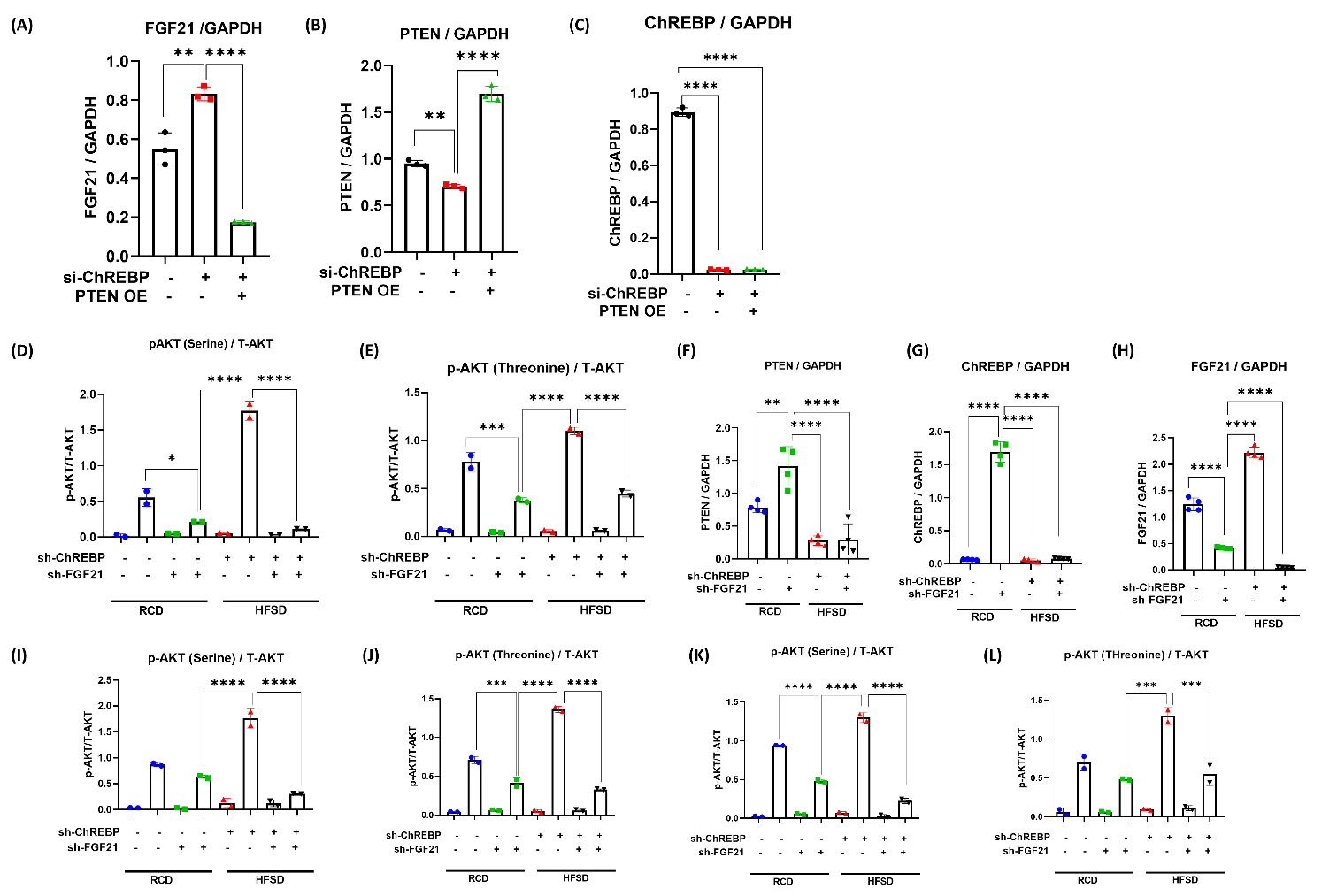


**Figure Supplementary 8: Quantification of Western Blot Images in Figure 3.** (A-C) Quantification of Figure 3B. (D-H) Quantification of Figure 3H. (I-J) Quantification of Figure 3J. (K-L) Quantification of Figure 3K. (I-J). Mean±SD. **P* < 0.05, ***P* < 0.01, ****P* < 0.001, *****P* < 0.0001.

**
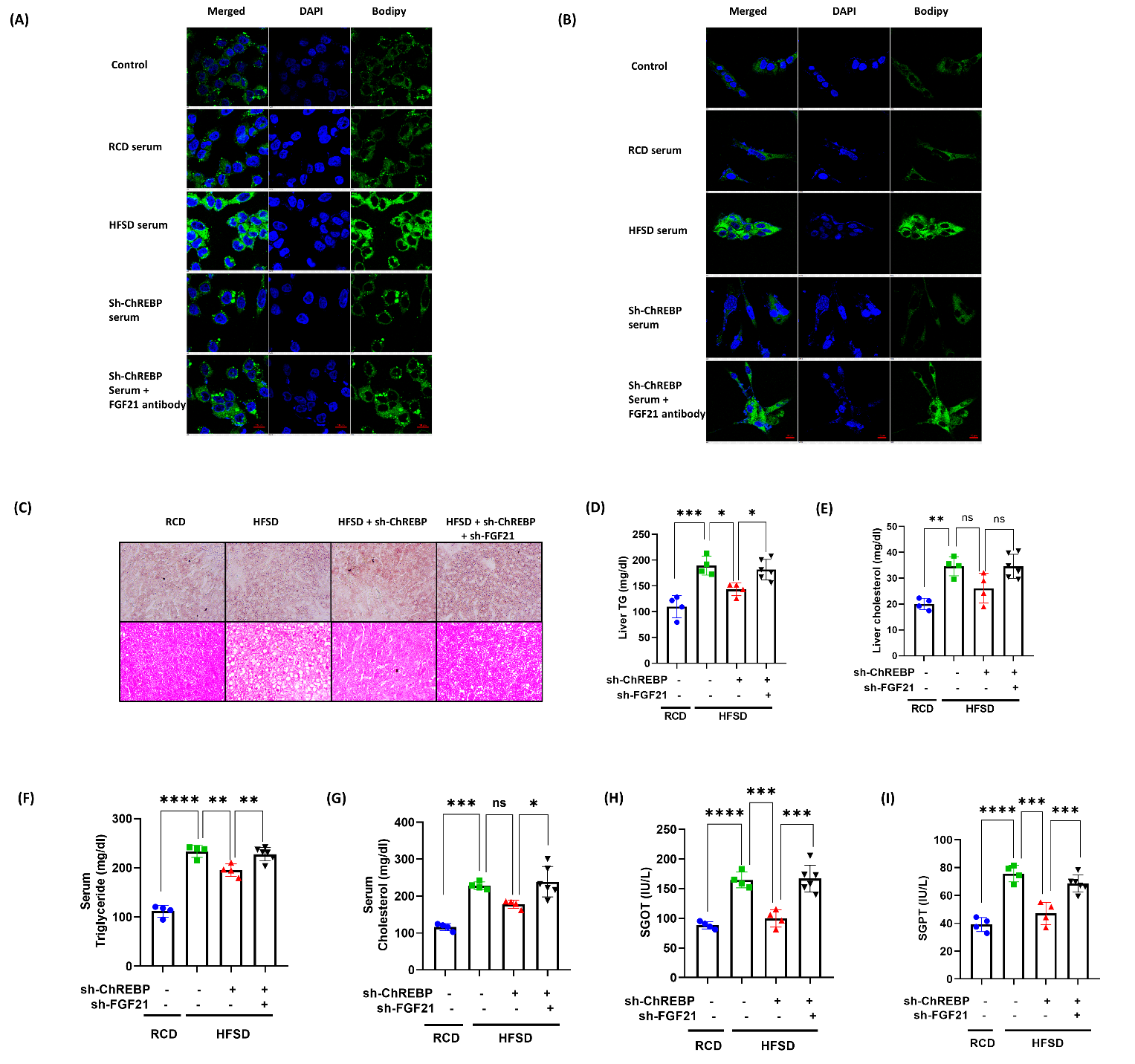
**

**Figure Supplementary 9: FGF21 and ChREBP double knockdown fails to attenuate hepatic DNL.** (A) Bodipy staining in HEPG2 cells, after treating the cells with RCD, HFSD+shScr, HFSD+shChREBP, and HFSD+shChREBP with FGF21 antibody treated serum. (B) Bodipy staining in 3T3L1 cells, after treating the cells with serum from RCD, HFSD+shScr, HFSD+shChREBP, and HFSD+shChREBP after neutralizing with FGF21 antibody. (C) ORO and H&E images of liver sections of said mice groups (40X magnifications). (D-E) Plot of liver triglyceride and cholesterol. (F-I) Plot the serum parameters like triglyceride, cholesterol, SGPT, and SGOT for all four groups. Figure A and B in this figure are in-vitro from HEPG2 and 3T3-L1 cells respectively. All other experiments from this figure are conducted in-vivo from C57BL6 mice. Mean±SD. **P* < 0.05, ***P* < 0.01, ****P* < 0.001, *****P* < 0.0001.


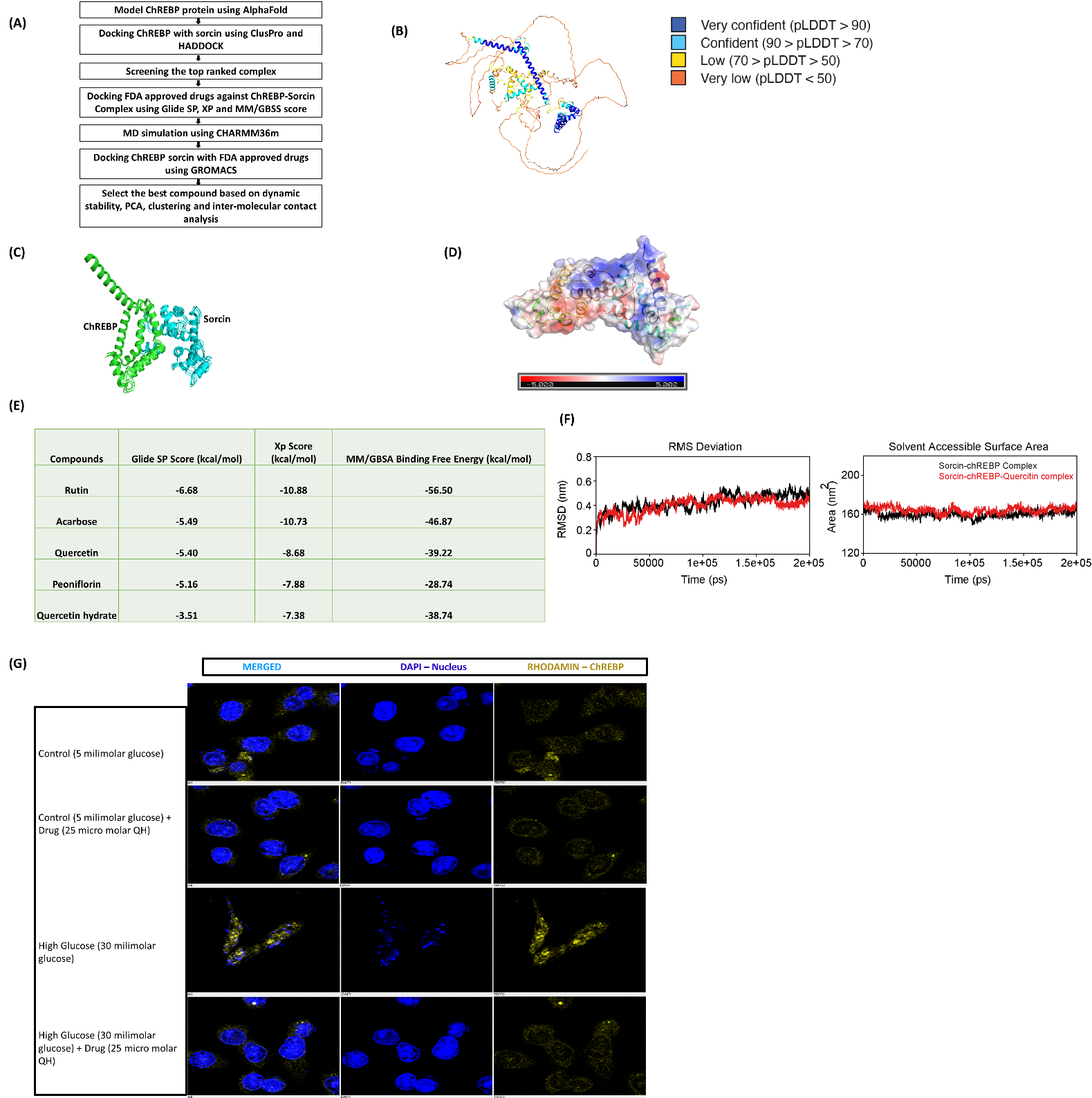


**Figure Supplementary 10: Quercetin can bind to ChREBP.** (A) The workflow for identifying QH molecule that binds to ChREBP. (B) AlphaFold structure of ChREBP with confidence scores. (C) Sorcin-ChREBP interaction structure. (D) Electrostatic potential map of sorcin-ChREBP interaction. (E) SP, XP, and MM/GBSA score of 5 FDA-approved drugs docked with sorcin-ChREBP complex. (F) MD simulation of sorcin-ChREBP interaction and sorcin-ChREBP-QH complex. (G) Immunocytochemistry image of HepG2 cells under low glucose, high glucose, and drug treatment conditions.


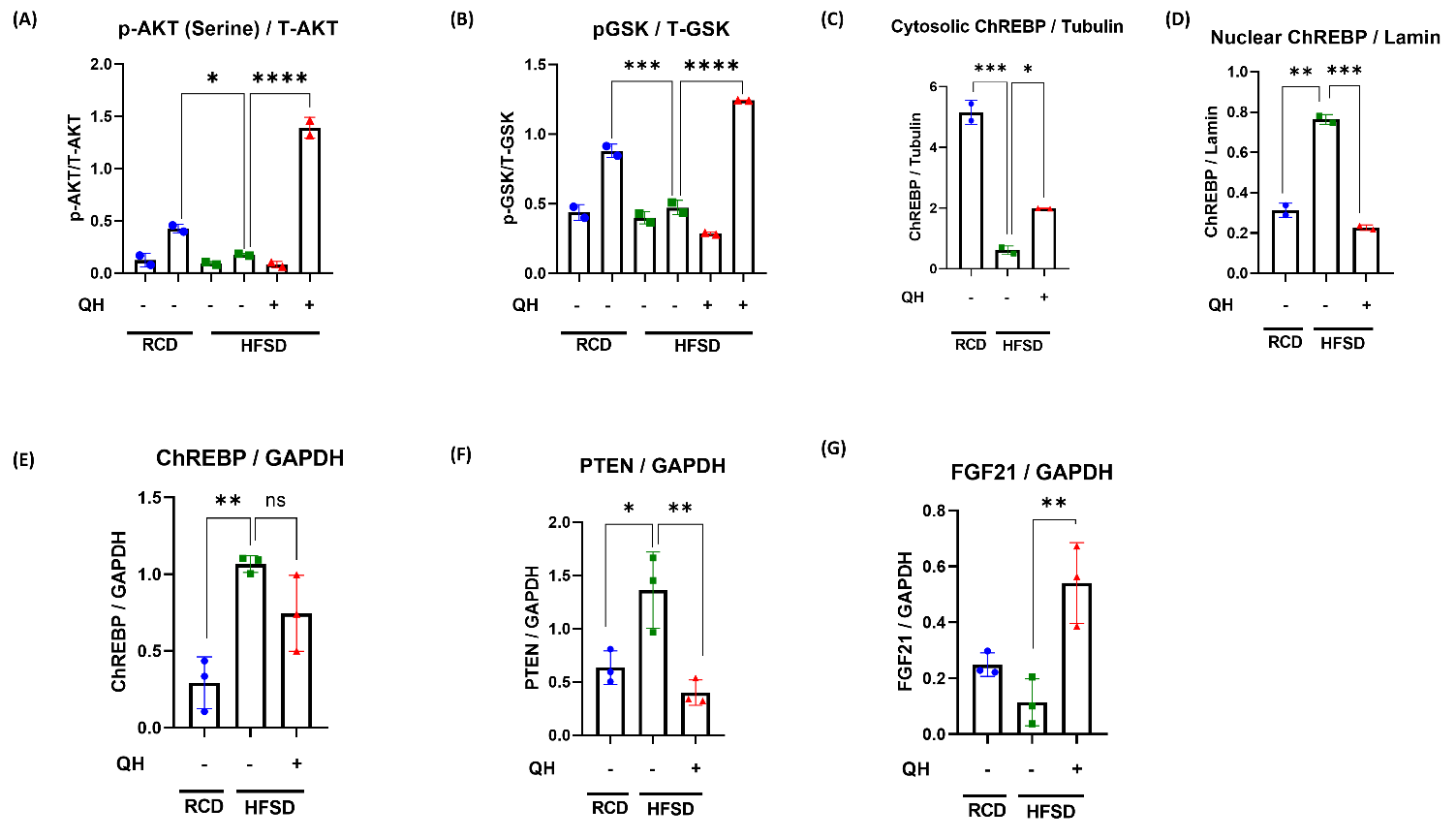


**Figure Supplementary 11: Quantification of Western Blot Images in Figure 4.** (A, B) Quantification of Figure 4H. (C, D) Quantification of Figure 4I. (E, F, G) Quantification of Figure 4J. Mean±SD. **P* < 0.05, ***P* < 0.01, ****P* < 0.001, *****P* < 0.0001.


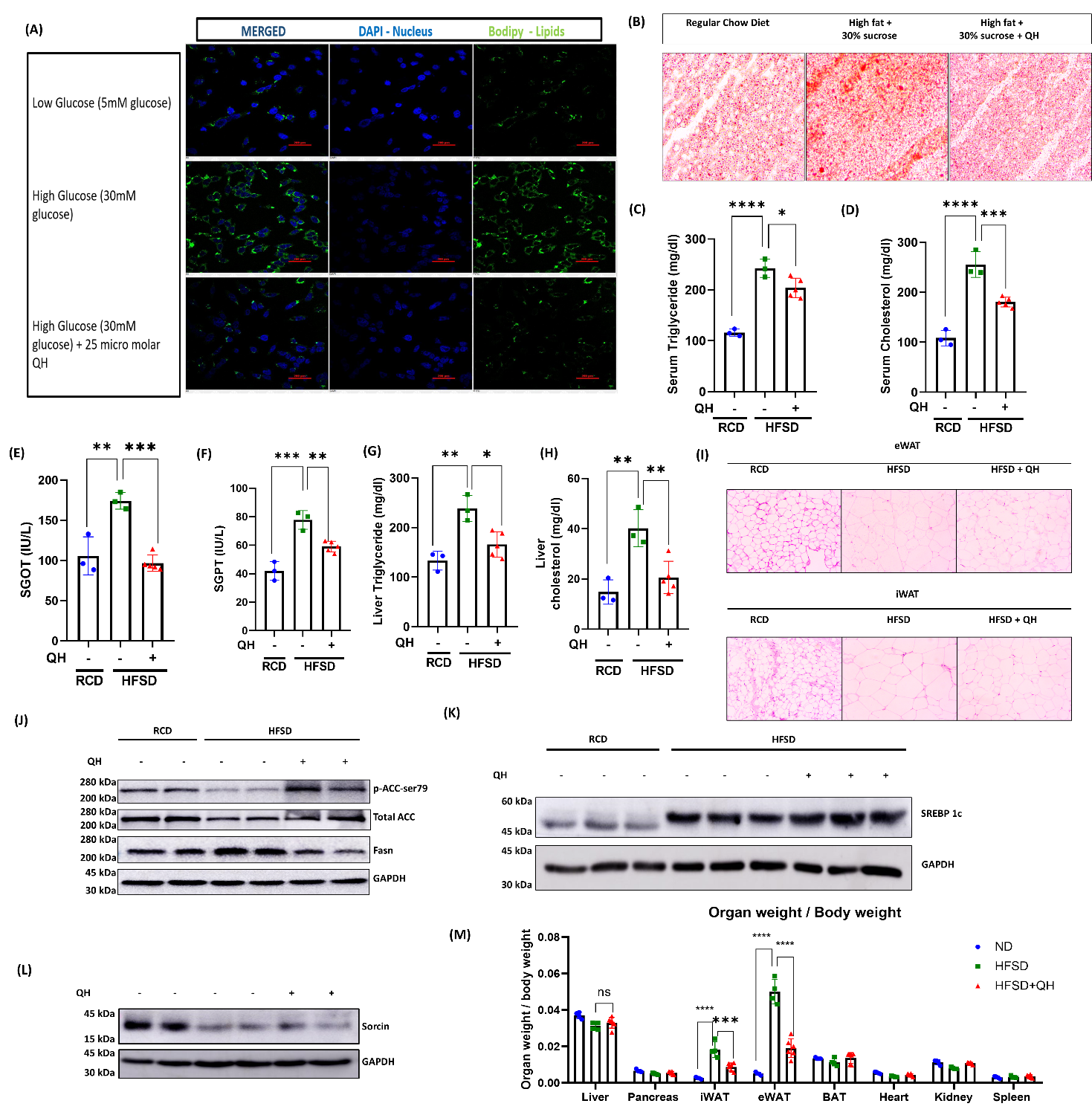


**Figure Supplementary 12: Quercetin administration can reduce hepatic DNL.** (A) Bodipy image of HEPG2 cells in low glucose (5mM), high glucose (30mM), and drug treatment. (B) ORO staining images of liver tissue were taken using a 10X lens. (C, D, E, F) Plot the serum parameters like triglyceride, cholesterol, SGPT, and SGOT for all four groups. (G, H) Plot of the liver triglyceride and cholesterol. (I) H&E staining of iWAT and eWAT; image captured in 10X lens showing hyperplasia and adipocyte size. (J) Immunoblot images and quantification of Fatty acid synthase (FASN), phosphorylated ACC serine (79), ACC, and GAPDH (used as loading control) from the mice groups as mentioned in Fig. 4C. (K, L) Immunoblot images and quantification of SREBP-1c and sorcin in the liver tissue samples of the same mice groups. (M) Relative organ weight of different organs from the tissue samples of the mice. Figure A of this figure is from in-vitro HEPG2 cells. All other experiments from this figure are conducted in-vivo from C57BL6 mice. Mean±SD. **P* < 0.05, ***P* < 0.01, ****P* < 0.001, *****P* < 0.0001.

**Supplementary Information**

**Chemicals:**

| S.No. | Chemical | Company | Cat. No. |
| --- | --- | --- | --- |
| 1 | iScript cDNA synthesis kit | Biorad | 170881 |
| 2 | iTaq Universal SYBR Green Supermix | Biorad | 1725121 |
| 3 | Insulin | Biorad | 10516 |
| 4 | gateway cloning system | Invitrogen | V49320 |
| 5 | lipofectamine 3000 | Invitrogen | L3000015 |
| 6 | lipofectamine RNAiMAX | Invitrogen | 13778150 |
| 7 | BCA assay | Thermo Scientific | 23227 |
| 8 | ORO | Sigma | O0625-25G |
| 9 | Bouin’s solution | Sigma | MFCD00146169 |
| 10 | Q5^®^ Site-Directed Mutagenesis Kit | NEB | E0554S |
| 11 | Bodipy 493/503 | Thermo | D3922 |
| 12 | Fluoroshield™ with DAPI | Sigma | F6057-20ML |
| 13 | Amicon Ultra-15 centrifugal filter | Merck | UFC901008 |
| 14 | Amicon Ultra-15 centrifugal filter 50 KD | Merck | UFC905008 |
| 15 | Nuclear cytosolic extraction kit | Thermo | 78833 |

# Antibodies:

| **S.No.** | **Antibody** | **Company** | **Cat. No.** |
| --- | --- | --- | --- |
| 1. | pAKT S473 | CST | 4058S |
| 2 | pAKT T308 | CST | 4056 |
| 3. | tAKT | CST | 9272s |
| 4. | pGSK3β | CST | 9323s |
| 5 | tGSK3β | CST | 9315 |
| 6. | pIRβ | CST | 3024 |
| 7. | tIRβ | CST | 3020 |
| 8. | PTEN | Abclonal | A11193 |
| 9. | Fgf21 | Abclonal | A23463 |
| 10. | pAMPKα | CST | 2535s |
| 11. | tAMPKα | CST | 2535 |
| 12. | pACC | CST | 3661 |
| 13. | tACC | CST | 3676 |
| 14. | GAPDH | CST | d16h11(5174) |
| 15. | ChREBP | CST | 58069S |

**Primers:**

| **Gene** | **Species** | **Sequence** |
| --- | --- | --- |
| ChoRE binding site of PTEN promoter FP | Human | CGGGGTACCTTTCCGAGGCGCCCTGCT |
| ChoRE binding site of PTEN promoter RP | Human | CCGCTCGAGATGGCTGCAGCTTCCGA |
| qPCR PTEN promoter ChIP FP | Human | TTTCCGAGGCGCCCTGCT |
| qPCR PTEN promoter ChIP FP | Human | ATGGCTGCAGCTTCCGA |

| S.No. | Plasmid | Cat.no, |
| --- | --- | --- |
| 1. | pChREBP Addgene | 39235 |
| 2. | pCMV-PTEN | 28298 |
